# On Residual Stress Development, Prevention, and Compensation in Metal Additive Manufacturing

**DOI:** 10.3390/ma13020255

**Published:** 2020-01-07

**Authors:** Kevin Carpenter, Ali Tabei

**Affiliations:** School of Mechanical, Industrial and Manufacturing Engineering, Oregon State University, Corvallis, OR 97331, USA; carpekev@oregonstate.edu

**Keywords:** additive manufacturing, residual stress, thermal stress, distortion, prevention, modeling, computation

## Abstract

One of the most appealing qualities of additive manufacturing (AM) is the ability to produce complex geometries faster than most traditional methods. The trade-off for this advantage is that AM parts are extremely vulnerable to residual stresses (RSs), which may lead to geometrical distortions and quality inspection failures. Additionally, tensile RSs negatively impact the fatigue life and other mechanical performance characteristics of the parts in service. Therefore, in order for AM to cross the borders of prototyping toward a viable manufacturing process, the major challenge of RS development must be addressed. Different AM technologies contain many unique features and parameters, which influence the temperature gradients in the part and lead to development of RSs. The stresses formed in AM parts are typically observed to be compressive in the center of the part and tensile on the top layers. To mitigate these stresses, process parameters must be optimized, which requires exhaustive and costly experimentations. Alternative to experiments, holistic computational frameworks which can capture much of the physics while balancing computational costs are introduced for rapid and inexpensive investigation into development and prevention of RSs in AM. In this review, the focus is on metal additive manufacturing, referred to simply as “AM”, and, after a brief introduction to various AM technologies and thermoelastic mechanics, prior works on sources of RSs in AM are discussed. Furthermore, the state-of-the-art knowledge on RS measurement techniques, the influence of AM process parameters, current modeling approaches, and distortion prevention approaches are reported.

## 1. Metal Additive Manufacturing and Residual Stresses

### 1.1. Introduction

Additive manufacturing (AM) is quickly becoming a leading method for manufacturing components across many industries, including automotive, medical, and aerospace [1]. Compared to traditional manufacturing, also called subtractive manufacturing, where components are fabricated by removing material from a larger stock, AM involves layer-wise addition of a material to form a three-dimensional (3D) component by fusion of the layers. Immediately, one can imagine the benefits of AM for very complex geometries or materials, like titanium alloys, which are strong and very difficult to modify with subtractive methods. While AM technologies exist for a variety of material systems, the focus of this paper is on metal AM, referred to simply as “AM”.

The costs to additively manufacture or 3D print a component may exceed that for traditional methods. For very simple geometries, it might be faster and easier to fabricate the part on a mill, lathe, or computer numerical control (CNC) machines. To 3D print a part, a computer-aided design (CAD) model must firstly be developed, and then sent to the AM machine, which slices the model into many very thin layers; then, each layer is deposited (either through powder or wire feedstock) onto the previous one and fused together, usually with the addition of heat. Comparisons were made for the cost of AM and traditional methods for several areas [1,2,3,4]; a general consensus is that, currently, traditional machining may remain more cost-effective than traditional machining in certain situations; however, as the technology is developing, AM is getting more efficient [2].

There are a few major obstacles preventing AM from fully surpassing traditional processes; AM is prone to microstructural defects and porosities that affect mechanical behavior of components, as well as residual stress (RS) formation, which can lead to geometric inaccuracies (part distortion) and deteriorate performance. Regarding geometrical accuracy, one must note that part distortions occur as a result of RS formation; the stresses that are generated tend to pull or push (depending on the direction of the RS) the material and deflect the part [5,6,7], as commonly seen in welding, hot rolling, and bending. In AM, deviations from the CAD geometry of as much as 2.1 mm were observed in a twin cantilever beam specimen of 11 cm by 1 cm [8,9]—an unacceptable amount of distortion for high-precision aircraft components. Regarding performance, RSs can adversely affect the structural reliability of the part in cyclic loading (fatigue) [10,11,12,13]. One study investigated RSs in electron beam AM (EBAM) titanium alloy, in as-built, stress-relieved, and hot isostatic pressed (HIPed) conditions, and the results indicated that hot isostatic pressing (HIPing) could cause microstructural changes to relieve RS and improve fatigue life of components, but neutron diffraction measurements suggested that most of the stresses were relieved during the EBAM build process at 600 °C. The improvements in fatigue life (over 100% between as-built conditions and HIPed, with respective fatigue strengths of 200 and 600 MPa at 10^7^ cycles) were attributed to other microstructure and porosity defects [14]. 

In AM, the multiple layers of material are fused together by the addition of heat. As the end of this section explains in more detail, this localized source of heat creates massive temperature gradients in the material, both in-plane in the newly added layer and through the thickness of pre-existing layers. The large thermal gradients are the primary source of RS formation in the part. Not only does each AM technology have unique features, e.g., material feedstock, heat source, and atmospheric conditions, as discussed in this section, but operator-input process parameters, such as scanning strategy (heat source path pattern), scanning speed, laser power, and build orientation can also lead to varying characteristics of RSs (size, direction, distribution).

RSs are defined as the stresses that exist within a body without any externally applied loads (i.e. the body is in equilibrium with its surroundings) [5,15]. They are also referred to as “internal” or “locked-in” stresses, and they can either strengthen a material, like toughened glass [16], or weaken a part. In AM, RSs might often be referred to as thermal stresses, since their origin is the steep thermal gradients in the manufacturing process. According to Shorr [17], thermal stresses are generated when a non-uniform temperature field causes localized thermal expansion that is interfered by non-expanding (or less-expanding) surrounding material, bodies, or parts. To fully grasp how RSs are formed, one must understand the underlying physics and governing equations for mechanical and thermal loads. Section 2 provides brief explanations of these concepts; for greater detail, the reader is referred to common engineering textbooks [15,17,18]. RSs are classified based on the size of their effects: macro- vs. micro-stresses [15]; or type I, type II, and type III stresses [5,12,13]. Type I stresses, or “macro” stresses, act over large lengths, with respect to the dimensions of the part. Type II stresses act over distances at the grain-size level and are often associated with phase transformations, while type III stresses are at the atomic scale (e.g., dislocation stress fields and crystal lattice defects) [12,13].

It was established that AM steel often exhibits a large portion of retained austenitic microstructure due to relatively rapid cooling [19]. However, the thermal stresses are believed to act as the driving mechanism for the phase formations of austenite to ferrite or martensite, in processes such as laser beam melting (LBM) [20].

**Figure 1:**
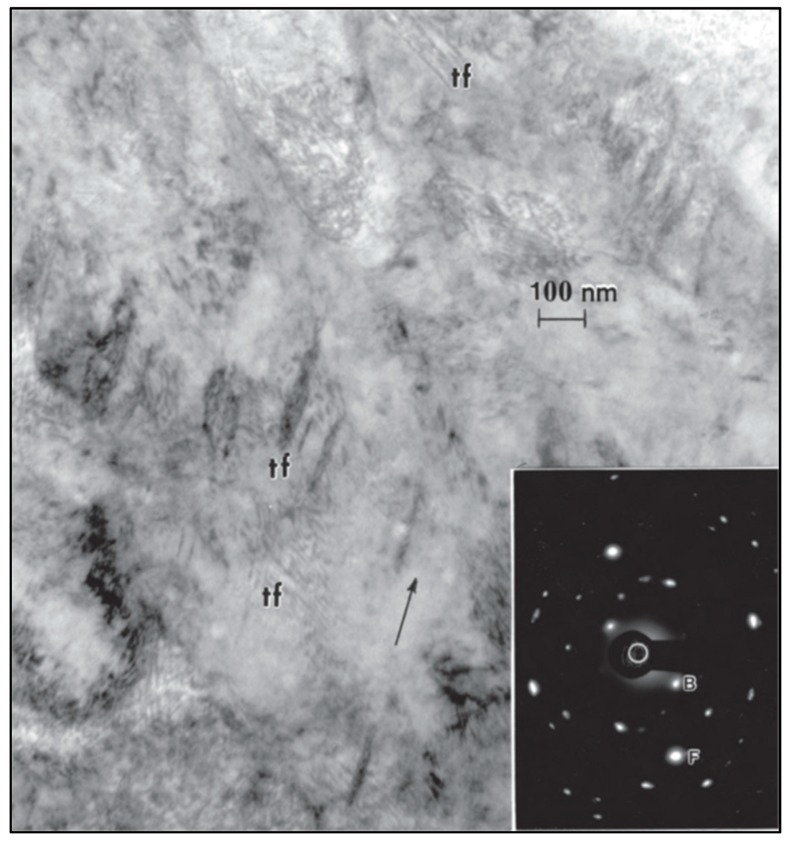
TEM image revealing dislocations, stacking fault traces, and deformation twin faults (tf) in austenite grains mixed with martensite in the build direction of selective laser melting (SLM) of a PH stainless steel. The black arrow indicates the ($\bar{1}12$) FCC direction, and the selected-area electron diffraction pattern image (bottom right) shows mixed diffraction spots (martensite: B = bcc-α, austenite: F = fcc-γ) [21].

Figure 1 shows a transmission electron microscopy (TEM) image of selective laser melted precipitation-hardened (PH) stainless steel, and it indicates that both martensitic and austenitic phases are present [21]. It was further demonstrated that a post-process heat treatment of LBM stainless-steel components partially transformed the austenite to martensite [20]. 

**Figure 2:**
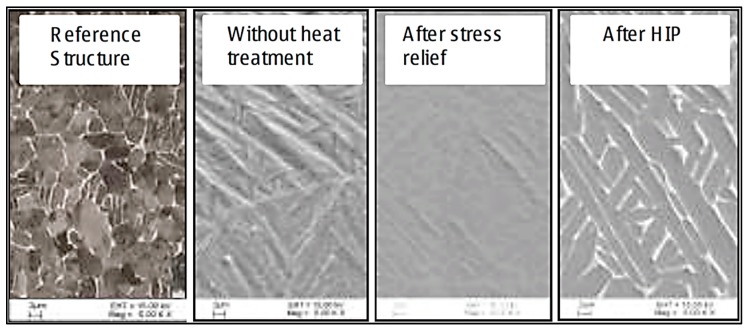
SEM images of Ti–6Al–4V microstructure for the reference structure (cast), and the SLM process without heat treatment, after a stress-relief heat treatment, and after hot isostatic pressing (HIPing) [22].

**Figure 3:**
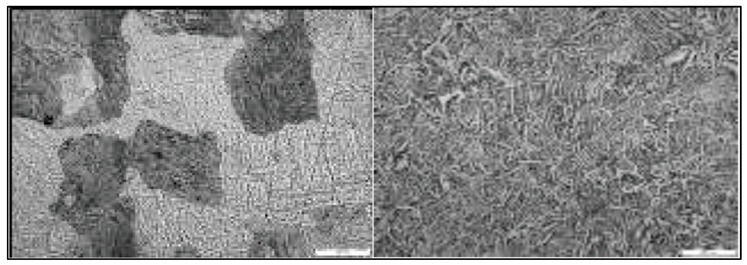
SEM images of Ti–6Al–4V microstructure of SLM specimens without a stress-relief heat treatment (**left**) and after HIPing (**right**) [22].

It was further shown by Uhlmann et al. [22] that a post-process stress-relieving heat treatment can change the microstructure in Ti–6Al–4V. It was observed that an inhomogeneous microstructure developed in the as-built SLM specimens. Yet, after HIPing or a stress-relief heat treatment, a more homogeneous microstructure developed [22], as shown in the scanning electron microscope (SEM) images in Figure 2 and Figure 3.

**Figure 4:**
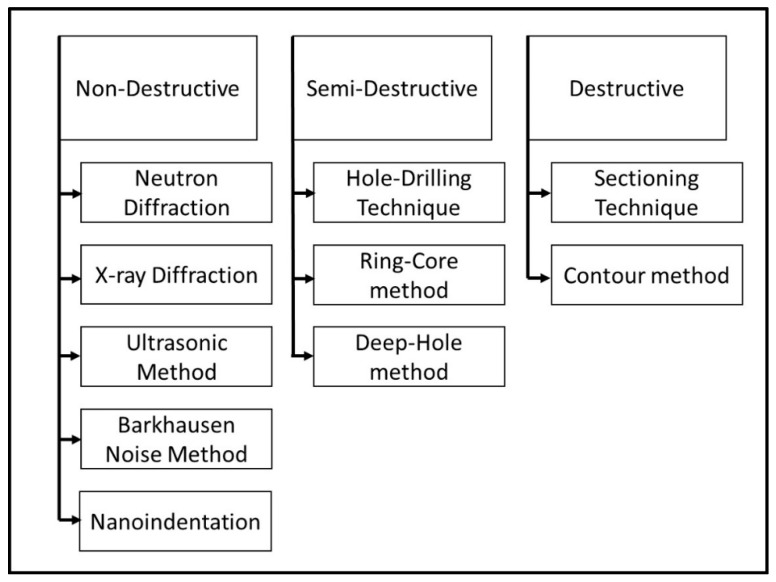
Categories of residual stress (RS) measurement techniques (adopted from Reference [13]).

Because of their impact on part performance, it is important to be able to experimentally measure or computationally model and predict RSs. Since RSs vary with respect to the size of the area over which they act, the selected measurement technique must have sufficient spatial resolution to capture the effects of the stress. Although stress is obtained indirectly from strain measurements, the methods are commonly referred to as residual stress measurements. A key concept for the determination of RSs is that the strains measured are elastic strains (i.e., stress and strain are related through Hooke’s law, discussed later). These strains can be measured either destructively (where the part experiences significant, irreversible alterations) or non-destructively (where the part retains most of its original integrity) (refer to Figure 4). An important effect that is the basis of measuring RSs destructively is that residual stresses, while forming, cause the part to distort, and, by removing the material which contains the RSs, the part relaxes to dimensions that would exist without RSs [12]. Section 3 provides summaries of experimental measurement of residual stresses. 

Because experiments can be costly, particularly for AM, it is often desirable to simulate a process numerically. A physically realistic and validated simulation can allow for rapid, inexpensive investigations into the individual effect of the technology features and process parameters on RSs in AM. Section 4 serves to report on modeling techniques in AM, with a focus on prediction of RSs. Finally, Section 5 discusses current reports on strategies to prevent RS formation and distortion mitigation in AM. One of the primary concerns is that many researchers are seeking to achieve the geometric accuracy by utilizing the effects of RS (shrinkage/warpage), but the RSs still play a detrimental role in mechanical properties and still exist in the part. As one can imagine, the challenges introduced above would prevent many parts requiring high-precision or structural integrity, such as aircraft components and medical devices, from being additively manufactured. These drawbacks were an area of investigation for many years, and this review paper serves to collect and report, specifically, on the most recent advancements toward RS prediction and prevention.

### 1.2. AM Technologies

**Figure 5:**
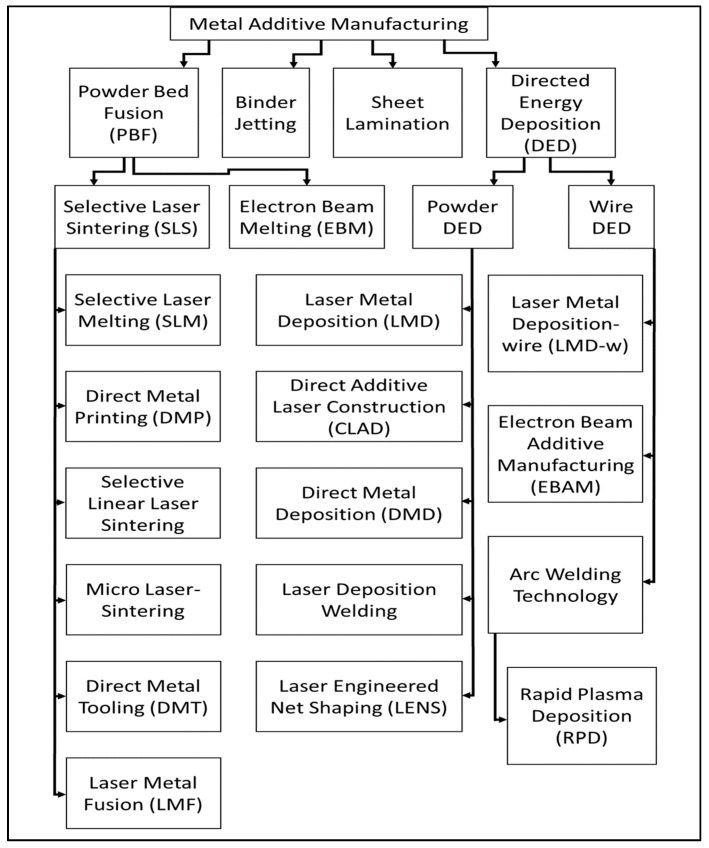
Categorization of the different additive manufacturing (AM) technologies (adopted from Reference [23]).

**Figure 6:**
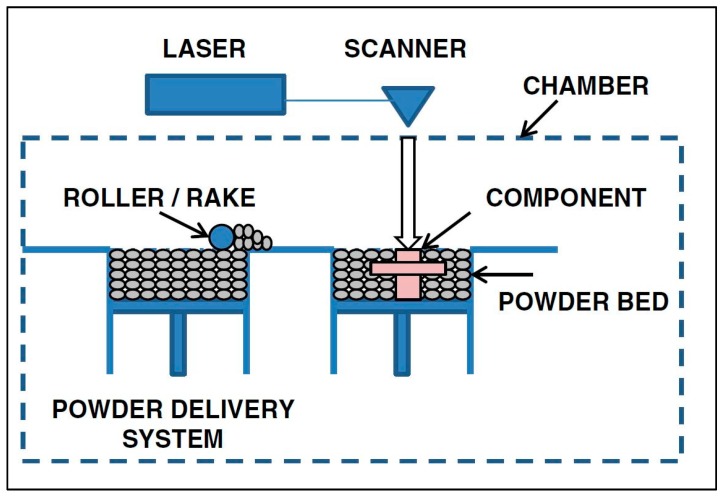
Schematic of typical powder bed fusion (PBF) technology [24].

**Figure 7:**
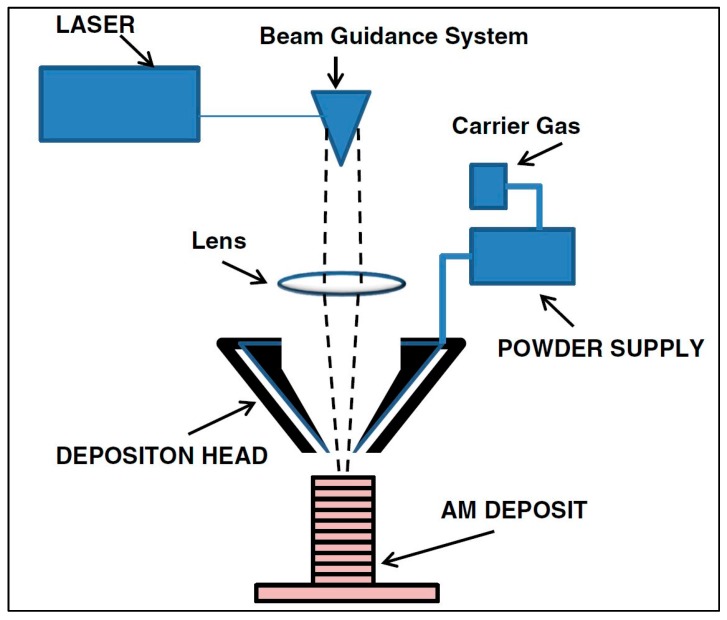
Schematic of typical directed energy deposition (DED) powder-based technology [24].

**Figure 8:**
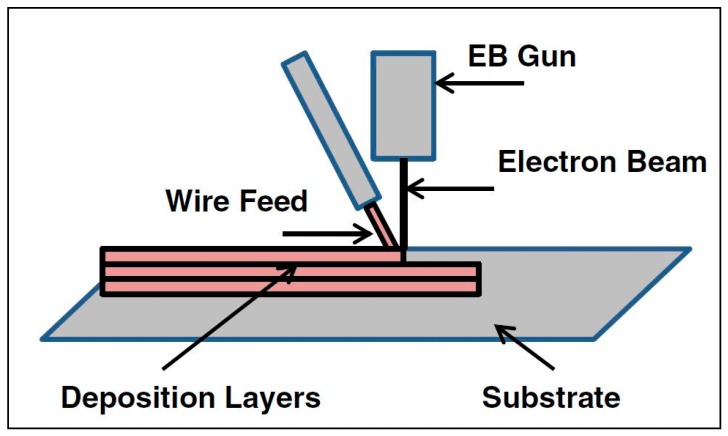
Schematic of typical DED wire-fed technology [24].

AM technologies are typically categorized by their feedstock and heat source. Figure 5 illustrates the various AM technologies, according to Nickels [23]. As seen in Figure 5, there are two primary categories: powder bed fusion (PBF) and directed energy deposition (DED). PBF encompasses those AM technologies that utilize a bed of powder particles; an arm called a “rake” or “roller” slides a thin layer of powder across the baseplate, and the layer geometry is scanned with a laser or electron beam, melting the powder particles into a solid layer, as shown in Figure 6. DED includes AM technologies that use either a powder or a wire feedstock; the powder is blown from a nozzle into the path of the heat source (a laser, typically), as seen in Figure 7, and the wire-fed AM technology is mostly related to welding processes. The wire material is fed and melted layer by layer, until the part is completely fabricated, as shown in Figure 8 [24]. The sections below discuss technologies that share common heat sources, as opposed to feedstock.

#### 1.2.1. Laser Melting

Application of a laser beam to melt the powder or wire feedstock is prevalent in AM; examples include selective laser sintering (SLS), selective laser melting (SLM), direct metal laser sintering (DMLS), laser-engineered net shaping (LENS), direct metal deposition (DMD), laser powder deposition (LPD), and selective laser cladding (SLC). The most common laser-based AM processes include SLS, SLM, DMD, and LPD [25].

In SLS, the bed of fine powders is heated to just below the material’s melting temperature, and a laser beam traces out the layer geometry, with sufficient power to sinter the powders to fuse together. SLM is very similar to SLS, except, instead of sintering the powder, it is fully melted. The difference between SLS and SLM is only a minor technicality, but the consequence is that the SLM laser source is usually of higher power [25]. 

In LENS, DMD, and LPD, instead of a *bed* of powder, a stream of powder material is delivered through a nozzle directed into the focused laser beam at the region of interest. LENS melts the metal powder that is delivered, usually by a pressurized inert gas, circumferentially around the laser head. DMD can either sinter or melt the powder, which is delivered through a number of nozzles in a similar fashion as LENS. The laser automatically positions itself to aim at defined points from a 3D model of the part. LPD also uses a stream of powder directed onto the part in the laser beam [25].

#### 1.2.2. Extrusion

**Figure 9:**
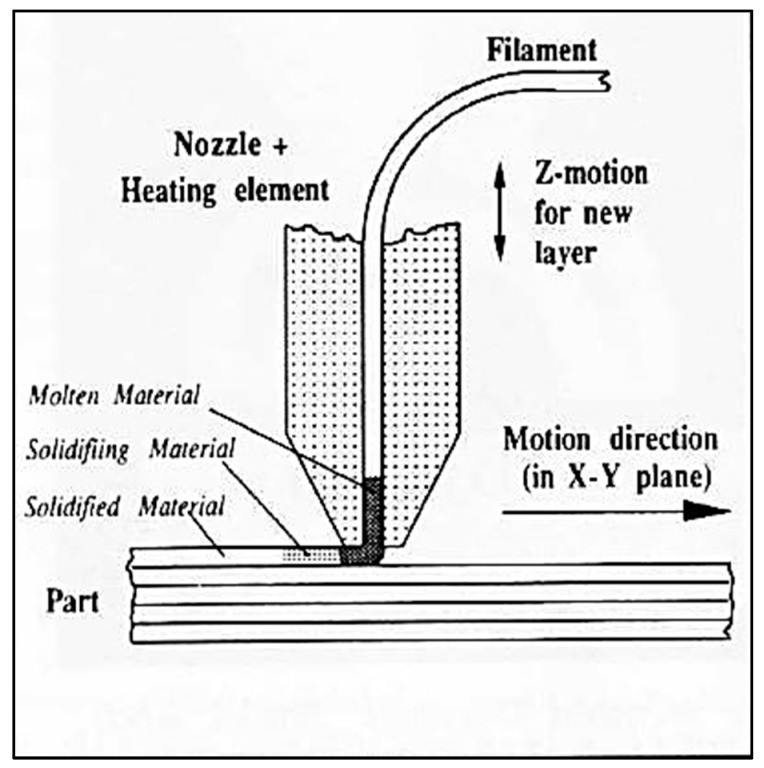
Illustration for a typical fused deposition modeling (FDM) process [26].

Extrusion processes do not involve materials in powder form; instead, the material is in the form of a wire. Typically, the nozzle through which the wire is fed is heated, and the material is softened or melted. From the nozzle, the material is deposited onto the build plate, layers are added, and they solidify upon cooling, as shown in Figure 9. 

FDM (fused deposition modeling) uses a moveable deposition head and deposits the wire material according to the computer-sliced 3D model. The heated extrusion nozzle generally heats the material 1 °C above its melting temperature, so that it solidifies immediately after deposition and fuses with prior layers. Typically, FDM has two deposition heads—one for the build material and the other for support structures [25]. 

#### 1.2.3. Material Jetting

Material jetting involves the controlled spraying of molten material or adhesive (called a binder), such that the particles bind to each other into a solid part. The binder holds the powder particles together, and no phase change occurs. 

#### 1.2.4. Electron Beam

Electron beam additive manufacturing (EBAM) is identical to the laser melting methods discussed above, except that the energy source is an electron beam and not a laser beam [27]. A difference from laser melting is that EBAM must be carried out in a vacuum chamber to avoid oxidation, thus restricting the size of the part to the vacuum chamber dimensions. EBM processes may also involve heating the powder bed during the build to reduce temperature gradients [28]. 

### 1.3. Sources of Residual Stresses in AM

**Figure 10:**
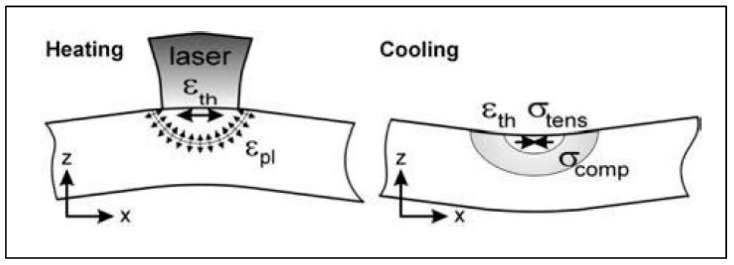
Illustration of the temperature gradient mechanism and distortion, where ε_th_ is the thermal strain, ε_pl_ is the plastic strain, and σ_tens_ and σ_comp_ are the tensile and compressive stresses, respectively [29].

As discussed previously, the various metal AM processes involve localized heating and cooling of top surfaces and the re-melting of preceding layers. As a consequence of these non-homogeneous thermal loads, RSs are generated, which may result in distortion of the part [5,6,7,28] and also deteriorate performance. As discussed by Mercelis and Kruth [29], Kruth et al. [30], Withers and Bhadeshia [5], and Withers [10], the main source of RSs in processes with the melting, solidification, and re-melting thermal cycle can be described by the temperature gradient mechanism (TGM) model. In the TGM model, the heat source is often a high-intensity point source, and the material temperature at the location of the heat source quickly elevates with respect to the surrounding material. The hot material expands, but is restricted by the less expanding, cooler material around it. This restriction creates a compressive stress in the heat source region. As the hot material cools, it contracts, but, again, the contraction is restricted by the less expanding surrounding material, which results in permanent tensile residual stresses in the part, and it often leads to a deflection or warped end product, as shown in Figure 10. 

The re-melting and re-solidifying of the pre-existing layers of material also contributes to the RSs in the part. When a new layer is deposited and melted, the recently solidified layers are likely to re-melt (depending on the process technology and parameters) or at least reach high temperatures again [31]. The previous layers cool and shrink underneath the new top layer. The shrinkage of pre-existing layers pulls and stretches the top layer, resulting in permanent tensile RSs. Another source of RSs in AM processes is the inhomogeneous lattice spacing. Because of the non-equilibrium process of AM, the microstructure is non-homogeneous. The inconsistent microstructure makes the lattice spacing spatially dependent, which makes the RS directions and magnitudes dependent on location [28].

**Figure 11:**
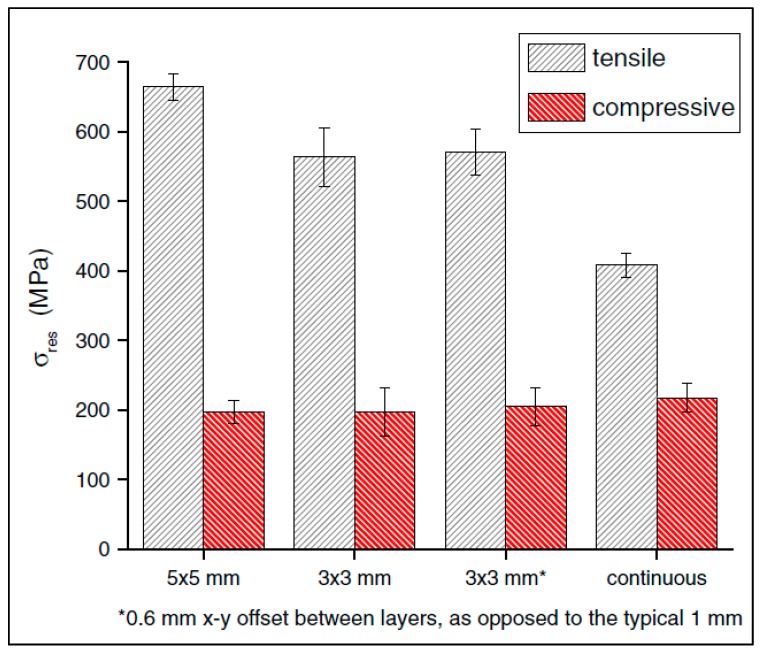
Measured residual stresses utilizing different scan island sizes [32].

Formation of RSs depends greatly on the AM process parameters. According to an investigation by Wu et al. [32] with stainless-steel 316L triangular prisms and L-shaped bars produced by SLM, various process parameters were studied and a reduction of RSs was achieved by decreasing the scan island size (discussed below) from about 650 MPa to about 400 MPa, as shown in Figure 11, and by increasing the laser power and speed; overall, compressive RSs typically existed in the center of the bar, and tensile stresses existed near the surfaces [32].

**Figure 12:**
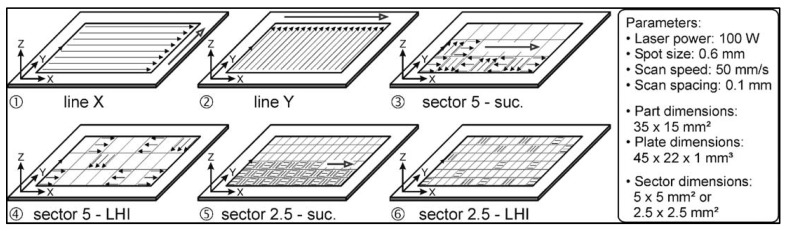
Scanning strategies described in Kruth et al. [30].

Kruth et al. [30] also investigated scanning strategy on RS formation and deformations during SLM of iron-based powders. Directional scanning, where the laser scanned the entire surface back and forth in one direction, resulted in large deflections in the perpendicular direction. Sector-wise scanning divided the surface into grid-like sections, called islands, and scanned each island either successively (adjacent grid sectors) or far apart to prevent heat influence from previous scans, called least heat influence (LHI), as shown in Figure 12. It was determined that the size of the islands did not greatly affect the distortion, but the successive scan strategy resulted in lower RS formation than both the directional scanning and the least heat influence sector scan strategy, likely due to the lower thermal gradients between the current island and the surrounding islands that were melted previously [30].

Lu et al. [33] investigated the island size on SLM of Inconel 718. It was concluded that the island size influenced the RS formation, contrary to the findings of Kruth et al. [30] for a different material. Island sizes of 2 × 2, 3 × 3, 5 × 5, and 7 × 7 mm were investigated. The 2 × 2-mm islands resulted in the lowest RS (around 100 MPa), but this was accompanied by the formation of cracks, which would have relieved much of the internal stresses. With consideration given to density, mechanical properties, and slightly lower RSs, the island size of 5 × 5 mm was determined to be optimal for Inconel 718 produced by SLM, yielding about 150 MPa [33].

Similar to Wu et al. [32], Mercelis et al. [29] showed theoretically and experimentally that, in SLM processes of stainless-steel 316L powder, tensile RSs formed at the top of the sample and the bottom baseplate interface, and compressive stresses formed in the center of the part. It was also determined that directional scanning resulted in high RSs in the transverse direction (about 110 MPa at the surface), and a small island, successive section scanning strategy resulted in low RS formation (about 65 MPa at the surface) [29], in agreement with the findings of Kruth et al. [30].

Liu et al. [34] also investigated the effects of energy input and scanning track length on RS formation in stainless-steel 316L bars produced by SLM. It was determined that compressive stresses existed in the center, while tensile stresses existed in the top layers. The energy input was controlled by the scanning speed; slower scanning speeds related to higher energy inputs. It was shown that the distribution of RSs was not affected (compressive in the center and tensile on the top), but the magnitude of the RSs increased with the energy input (slower scanning speeds generated larger RSs). For the greatest heat input, RSs of about 210 MPa were observed, while, for the lowest heat input, they were as low as 30 MPa. Finally, the length of the scanning track was investigated, leading to the conclusion that a longer track length led to larger RSs—nearly 200 MPa for the long track length, and as low as 45 MPa for the sector scanning. The relationship between track length and RS magnitude lies in the concept that the track shrinks in the scan direction upon cooling, and longer track lengths have less shrinkage compensation, leaving RSs [34].

According to van Belle et al. [35], powder thickness and cooling time between layers affects RS formation in a maraging steel produced by SLM. Consistent with previously discussed studies, tensile RSs were observed on the top layers. It was found that a thin, 20-μm layer of powder with a long cooling time (34 s) resulted in larger RS magnitudes (by three times) than samples produced with 40-μm-thick powder layers and 8-s cooling times between layers. In a study by Gusarov et al. [31], it was determined that RSs in SLM parts of alumina are dependent on the shape of the re-melted domain, but not the size. It was also modeled and experimentally shown that the maximum tensile RSs of about 75 MPa existed in the laser scanning (longitudinal) direction, and the transverse tensile stresses had about one-half the magnitude as the longitudinal stresses [35].

Wang and Chou [36] investigated the RSs formed in Ti–6Al–4V produced by EBAM and Inconel 718 produced by SLM. The material systems were of different geometries (and, thus, scanning strategies); therefore, a side-by-side comparison could not be made. The magnitudes of the RSs in the Ti alloy were reported as lower than the Inconel, which the authors attributed to the fact that EBAM takes place in a vacuum at high temperatures, resulting in a slower cool-down rate than SLM and, thus, stress-relieved components; furthermore, the EBAM process pre-heated the baseplate prior to melting the powder and, thus, had less steep thermal gradients. Another difference was that the titanium exhibited compressive RSs in the build and transverse directions, while the Inconel had compressive stresses in the transverse direction, but tensile stresses in the build direction. Stresses in the longitudinal direction were not reported. The reason that the Inconel had tensile stresses in the build direction was the unique scanning strategy; using 100 × 100-μm scan islands, the RSs were minimized, and the solidified islands pulled surrounding islands and previous layers in tension [36]. 

In a study by Cottam and Wang [37], H13 tool steel was fabricated using DMD and investigated for RS formation and microstructure characterization. It was determined that the RS distribution was inconsistent with other reports, attributed to the low-temperature phase transformation of the H13 steel. The RS in the top and bottom of the sample was reported to be compressive at 250 MPa, with a narrow band of tensile stress just above the central region in the build direction of about 150 MPa. Drawing parallels to welding processes, low-temperature martensitic phase transformations have compressive stresses, as shown in Reference [37]. 

The RS formation in FDM of acrylonitrile butadiene styrene (ABS) plastic was investigated by Saphronov et al. [6]. It was determined that, contrary to the literature, compressive stresses existed at the top and bottom of the specimen, and tensile stresses existed in the center. The disparity with the literature was attributed to the fact that the build plate used in this study was flexible, rather than rigid, which sets up an interesting question of the effects of build plate material on RSs in metal components [6].

## 2. Residual Stresses: Mechanics Background

The fundamental governing equation for elasticity (i.e., reversible deformation) is Hooke’s Law.

(1) $$\sigma_{ij}=C_{ijkl}\varepsilon_{kl},$$

where *σ* is the applied stress, *C* is the material’s stiffness matrix, ε is strain, and *i*, *j*, and *k* denote 1, 2, and 3, independently. In the 3D Cartesian coordinate system, 1 corresponds to the *x*-axis, 2 is the *y*-axis, and 3 is the *z*-axis.

In addition to mechanical loads, thermal loads can be related to strains in a body [17]. In this case, a change in temperature can cause a material to expand or contract, governed by the following equation:

(2) $$\varepsilon_{th}=\;\alpha \Delta T,$$

where *α* is the material coefficient of thermal expansion, Δ*T* is the change in temperature, and *ε_th_* is the thermal strain. The principle of strain superposition dictates that the mechanical strains (*ε*_σ_) and thermal strains ($\varepsilon_{th}$) are summed to a total strain value.

(3) $$\varepsilon =\;\varepsilon_{\sigma }+\varepsilon_{th}.$$

The total strain, given in the above equation, can used to obtain the stress in a part through the following constitutive equation [38]:

(4) $$\sigma_{ij}=\frac{E}{\left(1+\nu \right)\left(1-2\nu \right)}\left[\nu \delta_{ij}\varepsilon_{kk}+\left(1-2\nu \right)\varepsilon_{jj}-\left(1+\nu \right)\alpha \Delta T\delta_{ij}\right],$$

where *E* is the modulus of elasticity, *ν* is Poisson’s ratio, and *δ_ij_* is the Kronecker delta, taking values of 0 for *i* ≠ *j* and 1 for *i* = *j*.

## 3. Measuring Residual Stresses

### 3.1. Destructive Methods

Destructive measurement techniques are often referred to as stress-relaxation methods or mechanical methods. Common measurement techniques of this kind include hole-drilling, ring-core, deep hole, sectioning, and contour methods. By removing material which contains RSs and measuring the degree of the material relaxation (the deformation), the RS values can be determined.

#### 3.1.1. Hole Drilling

In a thorough report on measurement techniques for residual stresses [13], the hole-drilling method is described as the removal of material (a drilled hole) of relatively small size—typically on the order of 1.8 mm in diameter and 2 mm in depth—in the region where RSs are to be measured. Prior to drilling, strain gauges are arranged on the surface around the hole location (according to test standards), and, once the material is removed, the part relaxes, and the corresponding relaxation strains are measured; these strains are used to calculate the associated stresses [39].

This method is not very complex and offers fast results, making it a very common technique in practice. Note that the size of the hole is often not large enough to significantly impact the integrity of the part, and it can be repaired easily, if necessary. Issues exist, however, with this method, including the concerns of stresses induced by the machining process, a non-cylindrical hole, and non-circular (elliptical) shape. Despite these sources of error in the stress measurement, the hole-drilling method remains an established method for determining RSs.

#### 3.1.2. Ring Core

The ring-core method involves the measurement of strains of a surface induced by removing the material around the outside of it. If one considers the hole-drilling method, the ring-core method can be thought of as its inverse; a ring of material is removed to a certain depth, and the inner material is allowed to relax. Strain gauges on this inner material capture the relaxation strains. The strains are used to obtain the stresses associated with the relaxation. This method is superior to the hole-drilling method, because it offers much larger surface strains, but often causes significant damage to the part, which makes it far less desirable for use in practice. 

It is important to note that RSs may not be uniform through the thickness of the part; thus, many researchers employ an incremental hole-drilling or ring-core method. These methods remove material at incremental depths, so as to record stress values at various depths and build a stress profile through the thickness [13].

#### 3.1.3. Deep-Hole Drilling

Deep-hole drilling involves a combination of hole-drilling and ring-core methods. Firstly, a hole is drilled through the thickness of the part, and the diameter of that hole is accurately measured. The ring-core method is then introduced to remove an amount of material around that hole. The material between the ring and the hole relaxes as the RSs are removed, and the diameter of the hole is measured again. The change in diameter is used to calculate the stresses that were removed. Again, the incremental depth of the ring core in this method is used to build a stress profile through the thickness of the part. While this technique is largely destructive, it is found to be useful in instances where the part is thick and very large macro-stresses are expected to exist [13].

#### 3.1.4. Sectioning

Sectioning is a highly destructive method for measuring RSs. Sections of the specimen are removed, and the deformation is measured. It is important that the sectioning process be conducted without inducing plastic deformation or heat, which would interact with the RSs. As before, strain gauges are used on the specimen to measure the relaxation strains, which are used, in turn, to obtain the stresses [40].

The deformation that occurs as a result of the relaxation may be axial deformation or curvature; the axial deformation is a result of membrane RSs (surface stresses), and curvature is a result of bending RSs (through-thickness stresses). A common assumption is that the bending stresses vary linearly through the thickness [13]. 

#### 3.1.5. Contour

Finally, the contour method is a newer method for measuring RSs. In this method, the specimen is cut, and then the surface contour is measured, followed by data reduction and analysis. Arguably, the cutting of the specimen is the most important, since the quality of the cut (flatness, constant width, no discontinuities) influences the contour measurement and, thus, the data reduction and analysis steps. Commonly, a wire electric discharge machine (EDM), which uses sparks to remove material, is used in the material cutting process for uniform width and flat cutting. 

The contour measurement is carried out on the contoured surfaces created from the cut (contoured due to the relaxation from the removal of RSs). A coordinate measuring machine (CMM) is often employed in this step for high precision and accuracy. Data reduction is conducted by averaging each pair of points (the mirrored point on both cutting faces). Finally, the smoothed data (from the data reduction step) is used as an input (displacement boundary conditions) into a finite element model to calculate the original stress [13].

#### 3.1.6. Other Methods

Other destructive methods exist for measuring RSs: excision, splitting, and curvature (layer removal). Commonly used with thin plates, excision involves the removal of some material around a strain gauge to back out the stress in the part. Splitting is often used in thin-walled tubes, and it involves the sawing of a deep cut into the specimen, and the opening or closing of the cut by surrounding material (during the relaxation) can be related to RSs. The curvature method is often used in thin plates, where, by removing (or adding) a thin layer of material, the plate deflects upward or downward; the extent of the deflection is related to the RS within the part [13]. 

### 3.2. Non-Destructive Methods

The destructive methods discussed so far are advantageous because they provide drastic stress relaxation for measurements, but they are not ideal when dealing with parts for use. By definition, the induced damage degrades the integrity of the part and therefore, the part cannot be used for service after the measurement. There can be cases that the geometry allows for devising coupons to be separated from the functioning part, specifically for destructive RS measurement. On the other hand, non-destructive methods, where the part is not significantly altered, are much more favorable with expensive components. The non-destructive techniques include X-ray diffraction, neutron diffraction, and magnetic, ultrasonic, and thermoelastic methods.

#### 3.2.1. X-ray Diffraction

**Figure 13:**
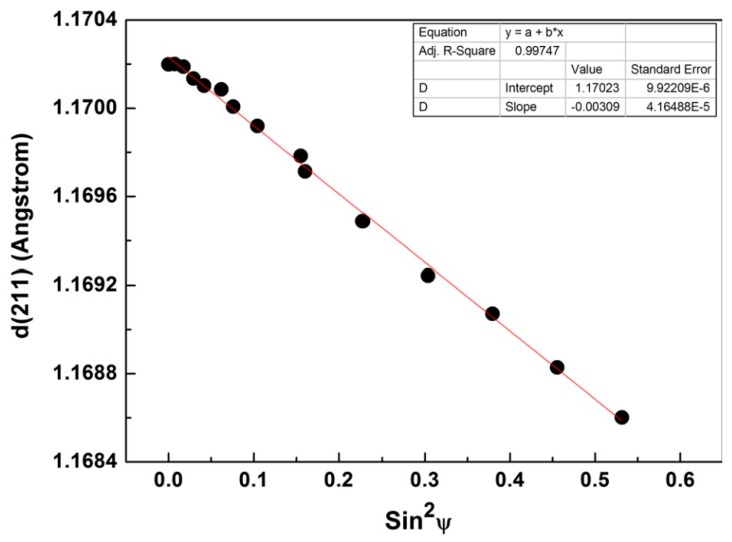
Plot of the interplanar spacing d for (211) planes vs. $sin^{2}\psi$ to obtain RS in H13 steel made by SLM [43].

X-ray diffraction (XRD) for measuring RSs makes use of the fact that, under stress, interplanar spacing in a crystal lattice can change [12,13]. In XRD measurements of RS, an X-ray beam is focused onto the sample, and the reflected beam is captured by a detector, which measures the intensity. Typically, the detector is moving with respect to the sample and X-ray source, capturing different reflective angles, and the XRD pattern of the sample is displayed as intensity (of the reflected beam) vs. twice the angle of reflection. By comparing the stressed XRD pattern to an unstressed pattern—typically a powder sample—and employing the Bragg’s law (see Equation (5)), the change in interplanar spacing can be related to elastic strains and, thus, to RSs, explained by the following equations [41]:

(5) nλ=2dsinθ,

and

(6) $$d_{\varphi \psi }=\left(\frac{1+\nu }{E}\right)\sigma_{\varphi }sin^{2}\psi -\left(\frac{\nu }{E}\right)\left(\sigma_{11}+\sigma_{22}\right),$$

where d represents the interplanar spacing, which corresponds to certain (hkl) planes or, equivalently, a specific 2θ Bragg angle. λ is the wavelength of X-ray beam, and n is the order of diffraction. $d_{\varphi \psi }$ denotes the stressed interplanar spacing for the same (hkl) planes, while the incident beam (or equivalently the sample) is tilted by ψ and rotated by φ. The elastic strain associated with the change in the interplanar spacing is directly related to the RS, $\sigma_{\varphi }$, which is normal to the (hkl) planes. Since other components of stress (e.g., $\sigma_{11}$ or $\sigma_{22}$) are not of interest for RS measurement, the slope of a plot of measured $d_{\varphi \psi }$ vs. $sin^{2}\psi$, yields $\left(\frac{1+\nu }{E}\right)\sigma_{\varphi }$, from which the RS, i.e., $\sigma_{\varphi }$, can be obtained straightforwardly if elastic properties of the material (*ν* and *E*) are known [41]. Measurement of RS by XRD is quite common for AM parts, as demonstrated by Mishurova et al. [42] and Yan et al. [43], amongst other researchers, and as shown in Figure 13.

A requirement of this method is that the material is crystalline; it must have many randomly oriented grains sufficient to produce an XRD pattern in any orientation of the surface. This method is often restricted to small geometries which can fit in the XRD machine, while also not interfering with the reflected beam to the detector. Furthermore, XRD is limited to measurements near the surface of the material (a few microns), and it cannot provide information regarding through-thickness stresses. This method could, however, be combined with some type of layer removal technique to generate the stress profile through the thickness, but it would then be considered destructive [13]. 

#### 3.2.2. Neutron Diffraction

Very similar to the XRD method is neutron diffraction. Changes in the lattice spacing due to elastic strains can be related to RSs within the material [13]. Neutron diffraction can be employed in different ways; a constant wavelength neutron source is used, and Bragg’s law determines the interplanar spacing from the diffraction patterns (similar to XRD); another option is to use a pulsed beam in conjunction with a time-of-flight method. The time-of-flight method holds the angle of incidence and reflection constant, but it varies the wavelength of the neutron wave; it shows precision measurements of Δd/d on the order of 10^−5^ for interplanar distances [44]. With the wide range of neutron energies (from the varied wavelengths), the neutrons with the highest energy arrive at the target specimen first. The strain is determined by the time between the source and detection as follows: ε = Δt/t [44]. An advantage of neutron diffraction over XRD is the greater penetration depth, as deep as 100 mm in aluminum and 25 mm in steel [13]. Major drawbacks are the size and cost of the equipment.

#### 3.2.3. Barkhauser Noise Method

Methods exist to determine RSs within ferromagnetic materials; one such method is the Barkhauser noise method. The theory behind this method is that the small magnetic regions within the material (which are magnetized along the crystallographic magnetization axes) have boundaries called domain walls. These domains are oriented with respect to one another such that the total magnetization of the material is zero (unless it is a permanent magnet). When an external magnetic field is applied to the ferromagnetic material, the total magnetization of the workpiece changes due to the rotation of the domains: the domains align parallel to the magnetic field direction. The movement of the domain walls is impeded by grain boundaries, dislocations, second-phase materials, and other impurities in the material. The restrictive forces on the domain walls from the defects and impurities can be overcome by applying a larger magnetic force. As the individual domains are suddenly rotated, the total magnetization also increases in jumps. The sudden increases in magnetization can induce electrical pulses in a coil. The Barkhauser noise is the combination of all of the electrical pulses from all of the domain movements. With an appropriate set-up, an alternating magnetic field can be generated, and stress can be determined from the magnetic force [12,13]. As mentioned, this method is only applicable in ferromagnetic materials, and it is really only useful for surface stresses (up to 0.2 mm on parts that were surface-hardened). While not as deep as neutron diffraction, magnetic methods also have greater penetration than XRD [12,13].

#### 3.2.4. Ultrasonic Methods

According to the acoustic elasticity effect, the velocity of an elastic wave propagating through a solid material has a dependency on mechanical stresses on the material. This effect allows for the RS measurement technique known as the ultrasonic method [12,13]. One common approach is the pulse-echo technique, where a transmitting transducer sends a wave through the material, and it detects the wave after it propagates through the material. The average of the RSs present in the material is determined from the time between pulse and detection. The biggest advantage of this technique over many of the others is its universality; this method is applicable to any solid medium. Also, the depth is on the order of millimeters (much deeper penetration than XRD), and the equipment is easy to set up, portable, safe (no radiation hazards), and inexpensive [12,13].

#### 3.2.5. Thermoelastic Methods

Understanding that deformation in a material can generate changes in temperature allows one to use temperature maps of a material to determine the stresses present. With the appropriate infrared camera set up, the temperature profile of a build can be obtained, and the internal stresses can be obtained. Because the temperature–stress effect is quite small, the resolution of infrared cameras must be fine enough for accurate determinations of stress, making this technique limited [12]. 

#### 3.2.6. Nanoindentation Techniques

Localized mechanical properties such as hardness and elastic modulus are influenced by RSs. Nanoindentation (NI) is a process via which a very small indent is made in the material while recording the applied force and penetration depth, in order to determine material properties. Many researchers used NI to measure the affected localized properties, and they calibrated the measurements to obtain localized surface RSs for polycrystalline materials and metallic glasses [45,46,47,48,49,50]. Suresh and Giannakopoulos [47] developed a standard method for estimating the surface RSs, and they showed mathematically and experimentally that tensile RSs allow for a larger contact area between material and indenter (and, thus, greater penetration depth) than compressive stresses for a given load. As described by Suresh and Giannakopoulos [47], an important consideration in using NI to measure RSs is that only surface/localized RSs can be measured, proportional to the depths and diameters of the indenter probes. Nevertheless, the NI methods remain effective non-destructive measurement techniques applicable to the surfaces of large components and thin films. Several research groups applied NI methods to AM parts for the determination of bulk material properties [51,52,53]. 

### 3.3. Common Residual Stress Measurement Approaches for Additively Manufactured Parts

Of the RS measurement techniques presented above, the non-destructive techniques are favorable because they do not cause major alterations to the build which degrade the part’s integrity; however, the destructive methods are the most standardized and allow for through-thickness stress measurements. While either approach can be applied to additively manufactured parts, residual stresses in these components are often measured non-destructively.

Stainless steel 316L fabricated by laser PBF was investigated by Wu et al. [32], and RSs were measured with neutron diffraction, coupled with the sectioning method. AM process parameters were investigated for their effect on RS formation. It was determined that compressive stresses existed within the center of the parts, and tensile stresses were observed at the surfaces [32]. 

**Figure 14:**
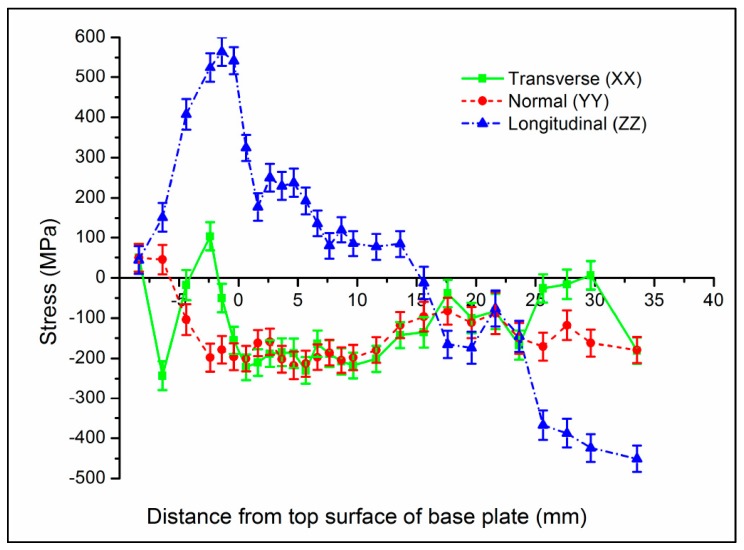
Residual stress measurements in wire-fed additive manufacturing of Ti–6Al–4V at the weld centerline distributed in the vertical *z*-direction [54].

The additive manufacturing of Ti–6Al–4V using a modified gas tungsten arc welding and an automated wire addition in a layer-by-layer process was investigated by Hoye et al. [54]. Residual strains were measured using the non-destructive neutron diffraction technique, and it was determined that, in the longitudinal direction (parallel to the weld direction), RSs were the most prominent, with values of 565 ± 35 MPa 1 mm below the surface of the baseplate. RSs of almost 70% of the yield strength of the titanium alloy were reported, and they were located near the surface at the centerline of the weld. Also, at the interface region between the deposited material and the baseplate, great variations in the principle stresses were observed [54]. An important consideration for this study was the fact that the build was machined after the additive manufacturing process to achieve the desired part geometry, which may have contributed to the magnitude of the RSs measured. The stress profile is illustrated in Figure 14 [54].

Ding et al. [55] manufactured a thin wall of mild steel using the wire and arc additive manufacturing (WAAM) process with a modified gas metal arc welding heat source. RSs in the sample were measured via neutron diffraction and the time-of-flight approach. An important finding was that the stresses in the longitudinal direction (parallel to the weld direction) were dominant over normal and transverse-directed stresses, with values as high as 450 MPa in the longitudinal direction. It was also observed that distortions and stress redistribution occurred after the sample was unclamped from the baseplate [55]. 

**Figure 15:**
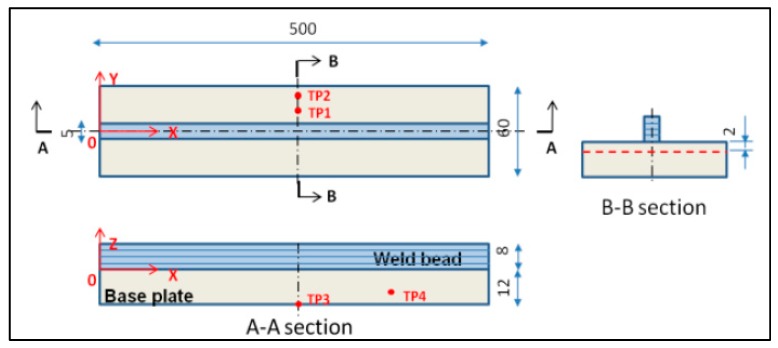
Geometry used by Ding et al. and Colegrove et al., with locations of thermocouple probes identified [55,56].

**Figure 16:**
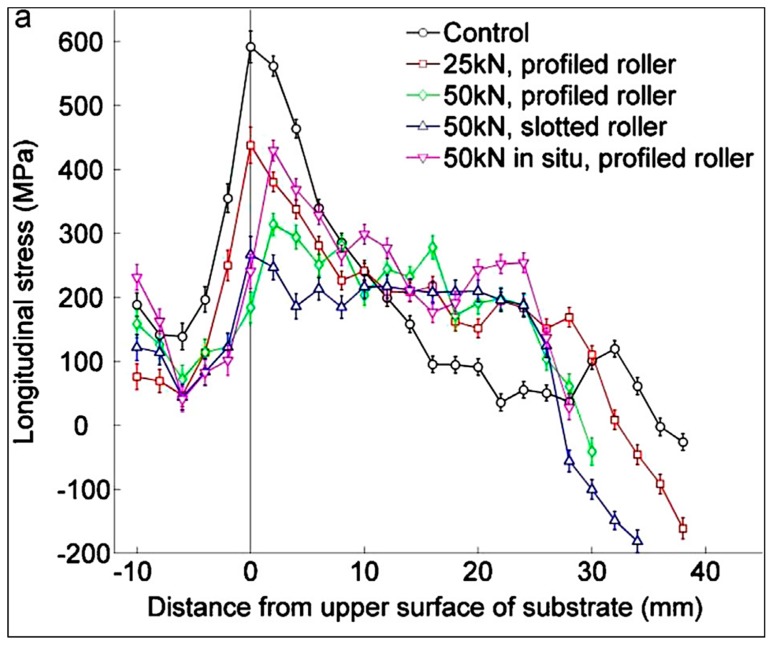
Longitudinal residual stress distribution through the depth of the sample prepared by Ding et al. and Colegrove et al. [55,56].

The WAAM process was also investigated by Colegrove et al. [56], who also used the neutron diffraction method to characterize the residual stress formed. Samples of mild steel were fabricated similarly to Ding et al. [55], as shown in Figure 15. After adding each layer, rollers were applied to relieve RSs and deformation; a profiled roller matched the surface shape of the deposited layer, and a slotted roller prevented lateral distortion. Similar results to Ding et al. [55] for the RS distribution were reported, reaching nearly 600 MPa [56] for the unrolled or as-built case, as shown in Figure 16.

Work was conducted by An et al. [57] using neutron diffraction methods for determining RS in Inconel 625 thin-walled, curved samples fabricated by laser PBF. It was determined that, at the top of the sample and near the baseplate, tensile hoop stresses of about 270 MPa existed. Also, tensile axial stresses of as much as 500 MPa existed around the edges, and compressive axial stresses of around 200 MPa existed in the middle of the sample. It was shown that the applicability of neutron diffraction was possible for Inconel 625 produced by laser PBF [57].

**Figure 17:**
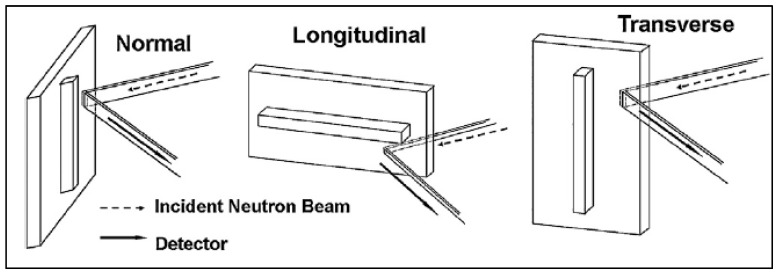
Geometry used by Cao et al., showing the neutron diffraction measurement orientations [38].

A titanium alloy, Ti–6Al–4V, was additively manufactured via EBAM by Cao et al. [38], and RSs were also measured with neutron diffraction; the geometry and measurement orientation are shown in Figure 17. As before, it was determined that the most significant RS was in the longitudinal direction, parallel to the beam path, reaching about 320 MPa. In the same work, the hole-drilling method was used and shown to be an effective method in additively manufactured parts [38].

Brice et al. [58] also used neutron diffraction to investigate 2219-T8 aluminum samples fabricated with EBAM. It was apparent that an RS concentration was located at the interface between the deposited material and the baseplate, reaching values of nearly 30 MPa along the entire interface. It was reported that the RSs were all compressive in the principle directions. It was also noted that, after the deposited material was un-clamped from the baseplate, the structure rebalanced, and the RS profile changed to accommodate the new equilibrium boundary conditions [58]. 

Simson et al. [59] explored RSs in SLM samples of 316L stainless steel. Using XRD, RSs were measured at the surface, and layers were removed via elecropolishing for additional XRD measurements at different depths. RSs measured on the top surface of the sample exhibited the largest magnitude in the scanning direction, and the largest RSs measured on the side of the sample were in the build-direction reaching about 220 MPa. The difference in the RS orientation for the different layers measured was attributed to the thermal gradient mechanism affecting the top surface residual stresses, and to the cool-down mechanism, affecting the build-direction stresses [59].

Ahmad et al. [60] investigated RSs in two materials manufactured by SLM: Ti–6Al–4V and Inconel 718. Through the contour method and numerical simulation, RS profiles were developed. The authors reported that significant distortion was observed in the titanium samples, but little noticeable distortion was seen in the Inconel. The RS distribution was similar between the materials, with the highest tensile RSs at the corners and surface of the specimens (titanium: 920 MPa, Inconel: 837 MPa) and compressive RSs at the center region (titanium: 335 MPa, Inconel: 459 MPa) [60]. 

An SLM titanium alloy (Ti–6Al–4V) was also investigated by Knowles et al. [61] with the destructive hole-drilling method. To capture the RSs through the thickness of the part, the hole was drilled incrementally, and the procedure followed the standard ASTM E837-08. The RSs measured in the samples were reported to be greatest at the surface, in some cases exceeding the material’s yield strength. The maximum measured value was 1508 MPa, and the minimum measured value was 135 MPa. The author concluded with the suggestion that work be performed to mitigate or relieve these stresses during manufacture [61].

In general, it is observed that, in AM components, tensile stresses are present on part surfaces, while compressive stresses exist in the center of the parts [29,30,32,34,60]. RSs of the greatest magnitudes are found in the direction of the scan line, compared to the transverse and build directions [38,54,55,56,59]. It is also frequently observed that the scan strategy affects RS formation, with small successive islands having lower magnitudes of RS than directional scanning [30,32,33]. Furthermore, the scanning speed was shown to affect the magnitude of the RSs; slower scanning speeds (and therefore greater energy input) resulted in larger RS than faster scanning speeds [34]. It was also observed that a large inter-layer dwell time results in larger RSs than with a short time between layers [35]. Finally, it was shown that pre-heating the baseplate of the build can reduce the thermal gradients and, thus, the RSs formed [36]. The EBAM process was suggested to generate RSs lower in magnitude, when compared to SLM, but a concrete comparison was not made; different geometries and scanning strategies were investigated between the AM technologies [36].

## 4. Computer Modeling of Residual Stresses

### 4.1. General Approaches for Modeling Residual Stresses

Essentially, to model RSs formed by AM processes, a thermal analysis is performed, followed by a mechanical analysis. The thermal analysis introduces the temperature distribution in the build, and the mechanical analysis determines distortions due to the thermal loads. These two frameworks are either coupled or uncoupled. If they are coupled, the mechanical analysis is performed at every time step of the thermal analysis, in order to account for the heat generated by deformations. The state of the system at each time step is calculated either implicitly or explicitly. In explicit methods, the current time step is used to calculate later time steps, and implicit methods use the current and the later state to determine the later state. Implicit methods can handle any time step size, while numerical instability may occur for explicit methods with large time steps. For highly non-linear processes, such as those in AM (temperature-dependent materials, plastic deformations, etc.), implicit methods may not converge, while explicit methods do, but they may require small time steps, increasing the computation cost. The non-linearity often forces an explicit approach, and convergence requires small time steps, resulting in large computational burdens for these types of simulations [62].

Zohdi [63] presented a computational methodology for the evolution of RSs from the deposition of hot particles, as seen in AM. The approach involved an implicit-staggered, coupled thermo-mechanical analysis, with an adaptive time step implemented and a finite difference time domain. The adaptive time step was to enhance computation time; small time steps were used when the system was changing rapidly, and larger time steps were used when the process was slower [63].

AM computer models are either at the micro or macro level, each of which provides different information. Megahed et al. [64] reported that micro-scale models of AM processes involve the interaction between the heat source and feedstock (powder particles, for example). The micro-scale models provide information about the melt pool size, temperature distribution, and material consolidation quality, whereas macro-scale models use the heat-affected zone dimensions and thermal cycle to calculate the RSs in the part. Typically, simulations performed for RS calculations are on the macro scale, and they assume some heat source distribution [65]. For the most physically accurate model of the AM process, one would model the heat source interaction with the feedstock at the micro level, and then model the fluid flow of the molten material, before finishing the simulation with a macro model for RS and part distortion prediction using the generated temperature distribution, as suggested by Ganeriwala [66] and Ganeriwala and Zohdi [67].

**Figure 18:**
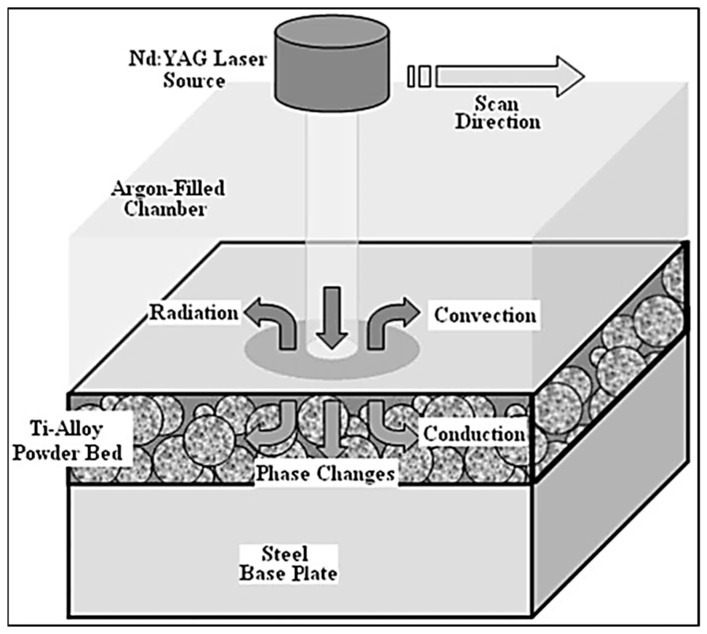
Laser melting of Ti alloy powder on a steel baseplate with heat transfer mechanisms shown [68].

Regardless of the spatial consideration of the model, the heat transfer in AM processes must be accounted for—the heat source for melting (laser or electron beam, typically), and the heat sinks for cooling mechanisms (heat dissipation through convection and radiation from free surfaces, as well as conduction through the material), as seen in Figure 18. 

No matter what modeling approach or time integration is used, conservation laws have to be obeyed and can be found in many heat transfer textbooks [69]. Generally, the energy balance for a closed system resembles the following Equation:

(7) $$Q_{flux}=Q_{cond.}+Q_{conv.}+Q_{rad.},$$

where *Q_flux_* is the total heat flux, and *Q_cond._*, *Q_conv._*, and *Q_rad._* are the conduction, convection, and radiation heat transfer mechanisms, respectively [68]. The heat conduction Equation can be expressed as follows:

(8) $$\frac{\partial }{\partial x}\left(k\frac{\partial T}{\partial x}\right)+\frac{\partial }{\partial y}\left(k\frac{\partial T}{\partial y}\right)+\frac{\partial }{\partial z}\left(k\frac{\partial T}{\partial z}\right)+\dot{q}=\;\rho C_{P}\frac{\partial T}{\partial t}\;,$$

according to Fourier’s law, where *k* is the thermal conductivity, *T* is the temperature, $\dot{q}$ is the rate of heat transfer into the system, *ρ* is the density, *C_p_* is the constant pressure heat capacity of the material, *x*, *y*, and *z* are the spatial coordinates, and *t* is the time. Enthalpy, *H*, is often taken into consideration to capture the phase-change information.

(9) $$dH=\;C_{P}dT.$$

Finally, in the case of heat input by the beam (modeled as a heat flux) and heat losses from convection and radiation, according to Labudovic et al. [70], the boundary conditions are treated as follows:

(10) $$k\frac{\partial T}{\partial n}-\dot{q_{s}}+h\left(T-T_{0}\right)+\sigma \epsilon \left(T^{4}-T_{0}^{4}\right)=0,\;$$

where *T*_0_ is the initial ambient temperature, *n* is the surface normal vector, $\dot{q_{s}}$ is the rate of heat input, *h* is the convection heat transfer coefficient, *σ* represents the Stefan–Boltzmann constant, and *ε* is the emissivity. The stress calculations are more or less the same throughout the literature, but the assumptions and simplifications made regarding heat flux, temperature distribution, and boundary conditions affect the RS predictions. Heigel et al. [71] stated that accurate RS calculations from computational models require detailed knowledge of the surface heat transfer; thus, any over-simplifications in the thermal analysis can result in inaccurate stress calculations. 

### 4.2. Specific Approaches of Modeling Residual Stresses in AM

The work of Ghosh and Choi [72] reported a methodology for the finite element analysis (FEA) prediction of microstructure formation, as well as RSs, in laser-aided DMD of H13 tool steel on a mild steel substrate. Using the same heat conduction equation and boundary conditions described by Equations (8)–(10), a transient thermal analysis was performed, assuming a Gaussian heat flux distribution, given by the following equation [73]:

(11) $$Q=\frac{AP}{\pi r^{2}d_{0}}e^{\left[-2\frac{\left({\left(x-v_{x}t\right)}^{2}+{\left(y-v_{y}t\right)}^{2}\right)}{r^{2}}\right]}\frac{1}{5}\left[-3{\left(\frac{z}{d_{0}}\right)}^{2}-2\frac{z}{d_{0}}+5\right],$$

where *A* is the laser absorption coefficient of the powder, *P* is the laser power, *r* is the laser spot radius, *d*_0_ is the penetration depth, *x*, *y*, and *z* are the coordinates of the laser spot center, *v_x_* and *v_y_* are the laser spot speeds in *x-* and *y*-directions, respectively, and *t* is time. The temperature results were provided to a user-defined subroutine to determine the fraction of each phase of the material; strains from transformation plasticity and volume change were also computed. The uncoupled mechanical analysis then computed elastic, plastic, and thermal strains at each time step. It was reported that the time steps were much smaller during the rapid initial heating than for the slow cooling process in order to converge. It was also stated that the time steps for the thermal and mechanical analyses were independent of each other. The stresses generated were obtained from the calculated strains, assuming a linearly elastic and linearly plastic relationship. Experiments were carried out, and RSs were measured with XRD. Discrepancies between experiment and simulation were attributed to X-ray beam size relative to the interface size between laser passes, as well as the formation of “peaks” and a “valley” due to the overlap between laser passes; the XRD likely missed data points in the “valley” [72]. 

An investigation by Zaeh et al. [74] explored the transient physical effects in SLM processes for 1.2709 tool steel. Two types of coupled thermo-mechanical simulations were carried out: a layer-based detailed model and a part-based global model. The layer-based model utilized sufficiently accurate heat source models that mimicked the thermal interaction between the laser beam and the powder bed. This model allowed for process parameter optimization (scan strategy and speed, as well as baseplate temperature) regarding RS and deformation on single layers. The part-based global model allowed for the entire part quality. The heat source was applied uniformly to entire layers, without concern for the scan strategy. The part-based global model calculated maximum tensile residual stresses of about 1000 MPa in the horizontal plane and 416 MPa in the build direction, and maximum compressive stresses of 1100 MPa in the horizontal plane and 804 MPa in the build direction at the outer supports; the layer-based model predicted tensile stresses of 86 MPa in the longitudinal direction and compressive stresses of about 628 MPa. Experimental validation of the simulations using neutron diffraction revealed tensile stresses of 305 MPa in the longitudinal direction at the top surface and a compressive stress of 184 MPa at the bottom of the part. The predicted stresses in the longitudinal direction were about 187 MPa at the top surface of the part. The simulations predicted deformations and RSs that were within measured values [74]. 

Zaeh and Branner [75] extended the work of Zaeh et al. [74] by investigating RS and deformation in SLM of 1.2709 tool steel through simulations. A coupled thermo-mechanical system was employed, and calculated RSs were verified with neutron diffraction. Simplifications were made to prevent long computation times; for instance, the thermal load was applied to an entire layer for 20 ms, instead of modeling the individual scanning vector. Radiative and convective boundary conditions were applied to the part during the cool-down phase, as well as conduction to the baseplate. Tensile stresses were calculated within the horizontal plane to have a maximum of 1000 MPa at the edge of the structure and 416 MPa in the build direction. For a specimen fabricated with the parameters used in the model, the experimentally measured RSs were lower than the simulation predictions. Discrepancies were attributed to the simplifications of the model and the differences in the support structure geometry between model and experiment [75]. 

**Figure 19:**
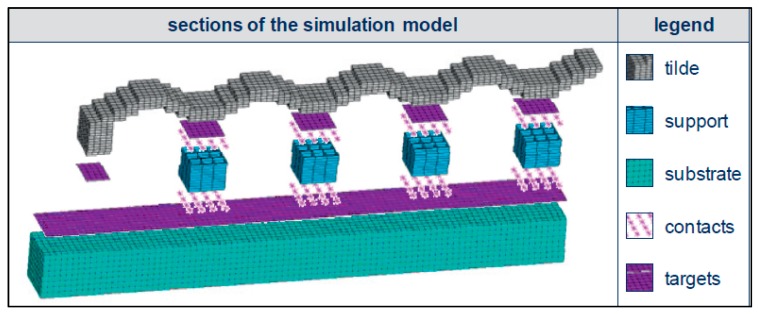
Geometry used by Krol et al. for finite element simulations [76].

Krol et al. [76] compared simulation results to experimental measurements of RSs formed in AM processes; the geometry can be seen in Figure 19. The heat flux was applied to the entire layer, without modeling scan strategies. An experimental investigation of process parameters on residual stress formation revealed, with neutron diffraction, that the finite element model must be of sufficient detail to capture RS progression as a function of process parameters [76].

The SLM of titanium and nickel powder was simulated by Gu and He [77] for the purpose of RS predictions. The calculated stress values were compared to qualitative experimental results. The simulation presented was a coupled thermo-mechanical analysis with a Gaussian heat source distribution, and the results indicated that the maximum RSs occurred at the end of the scanning track, with values of 86.3 MPa in the scanning direction, 95.7 MPa in the transverse direction, and 23.2 MPa in the build direction. The qualitative experimental results were based on visual crack formation in the manufactured part, indicating locations of large RSs, which agreed with simulated stress distributions [77].

EBAM was simulated by Cao et al. [38] for the purpose of predicting RS and part distortion. Experimental measurements using neutron diffraction and the hole-drilling method were used to validate the simulations for Ti–6Al–4V specimens. After simulating different types of heat sources (Gaussian heat distribution, simple point heat source, double ellipsoid, and uniform heat source), a uniform heat distribution most closely fit the shape of the molten pool in the experiments. The temperature distribution was obtained through the heat conduction equation and heat transfer equation discussed previously. To account for the effect of fluid flow of the molten material on heat transfer (the Marangoni flow), the thermal conductivity was artificially increased by a factor of three, based on other reported works [78]. The RSs were calculated in a coupled mechanical analysis. RSs were measured at five points in the longitudinal direction and five points in the transverse direction, showing maximum values of about 320 MPa; comparing the measured values to the simulation, good agreement was found, but it was noted that five data points were insufficient to fully validate the model. It was also noted that the model predicted zero distortion near the plate extremities, but measurements with a coordinate measurement machine (CMM) indicated small distortions [38]. A final comment indicated that the effects of microstructure evolution were not simulated, which would have impacted the predicted RSs, as explained by Myhr et al. [79].

**Figure 20:**
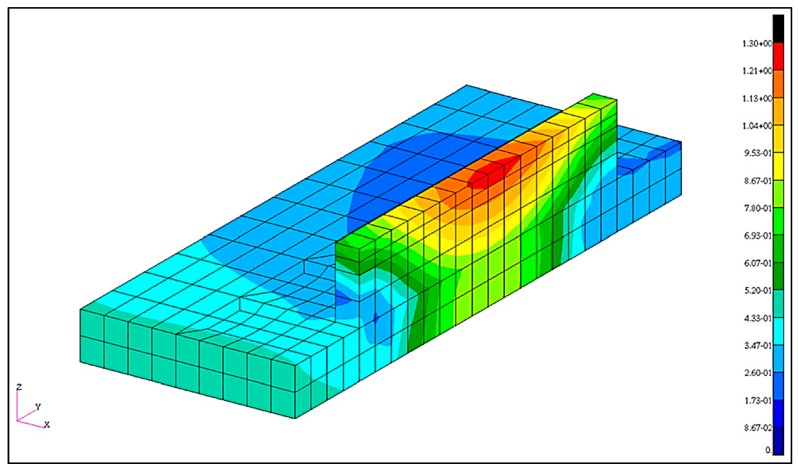
Displacement magnitudes (in mm) at the end of the simulation: maximum displacement of 1.3 mm, minimum displacement of 0 mm [80].

Denlinger et al. [80] modeled an electron beam-deposited Ti–6Al–4V AM part, and they explored RSs generated. The uncoupled thermo-mechanical analysis assumed a Goldak double ellipsoid heat source distribution [81], given as follows:

(12) $$Q=\;\frac{6\sqrt{3}P\eta f}{abc\pi \sqrt{\pi }}e^{-\left[\frac{3x^{2}}{a^{2}}+\frac{3y^{2}}{b^{2}}+\frac{3{\left(z+v_{w}t\right)}^{2}}{c^{2}}\right]},$$

where *P* is the power of the electron beam, *η* is the absorption frequency, *f* is the process scaling factor, *x*, *y*, and *z* are the coordinates, and *a* is the transverse dimension, *b* is the melt pool depth, and *c* is the longitudinal dimension of the ellipsoid, *v_w_* is the scanning speed, and *t* is the time. Distortion measurements were compared against the simulated distortion, and the magnitudes of the simulated distortions were larger than those measured, but agreed reasonably well with a maximum error of 29%. The magnitude of the displacement predicted by the simulation are shown in Figure 20 [80].

A finite element method was developed by Ding et al. [55] for the thermo-mechanical response during a WAAM process of mild steel, employing a steady-state thermal model, which was compared to a conventional transient model and experimental measurements of residual stresses. In both models, the thermal and mechanical systems were coupled, and the Goldak double ellipsoid heat source [81] was applied. The models did underestimate the value of RSs in a one-layer wall, which was attributed to the microstructure evolution not being captured by the model. Overall, the steady state and thermal models agreed well with experimental measurements of residual stress, with the steady-state model showing an 80% advantage in terms of computation time [55].

Chae [82] investigated RS evolution in DMD of low alloy steel by numerical simulation of laser–powder interaction and molten fluid flow, and then the temperature distribution was used to calculate the thermal strains, volumetric strains (from martensitic phase transformations), and plastic strains due to the phase transformations. The strains were used to calculate RSs from three-dimensional Hooke’s law. XRD was used to measure residual strains for a comparison to the simulation predictions, and it was determined that the stresses at the top surface were over-predicted by 8.6%, and those at the melt pool interface were over-predicted by 35.7%. The discrepancies were attributed to the extrapolated values of AISI 4340 steel material properties at high temperatures, due to limitations of access in the database [82].

Parry et al. [83] explored the effect of scan strategy on RS formation through simulations of SLM of Ti–6Al–4V alloy. The coupled thermo-mechanical analysis utilized the Goldak double ellipsoid heat source model, and time-independent plasticity was modeled with a von Mises yield criterion to capture the cyclic non-linear work hardening effect (the Baushinger effect). It was not explicitly stated whether or not microstructure evolution was modeled [83]. 

**Figure 21:**
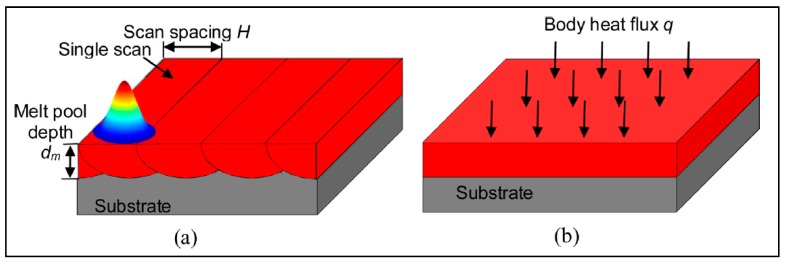
Micro-scale simulation of scan strategy (**a**), to obtain an equivalent layer heat flux (**b**) [9].

Li et al. [9] developed a multiscale finite element approach for prediction of part distortion and RSs generated during SLM of AlSi10Mg powder. The proposed simulation involved the micro-scale modeling of a single track to obtain a temperature history, and then a meso-scale model, which extended the micro-scale model to a deposited powder layer, as shown in Figure 21; finally, a macro-scale model was used to apply the thermal load calculated from the meso-scale model to an entire part. Applying the Gaussian heat source distribution to the surface of a powder layer, the micro-scale model was solved. The temperature history was used as an input to the meso-scale model, where an equivalent body heat flux was applied to the entire layer as follows:

(13) $$q=\;\frac{AP}{d_{s}d_{m}H}\;,$$

where *d_s_* is the laser spot diameter, *d_m_* is the melt pool depth, and *H* is the scan spacing (hatch spacing). Finally, the macro-scale model was a coupled thermo-mechanical analysis, and the part was divided into 12 layers, with each layer given the equivalent body heat flux [9]. 

Li et al. [84] simulated SLM of iron-based powders to predict RSs and part distortion. Due to a lack of availability of material properties, temperature-independent properties were used in the model. The thermal loads were similar to those described in Li et al. [9]. Comparisons to experimental data were not discussed for model validation, but it was suggested that temperature-dependent material properties would be investigated in future works [84].

Denlinger et al. [85] modeled an EBAM process of Ti–6Al–4V. RSs and distortions were calculated and compared to measured stresses from the hole-drilling method. The thermo-mechanical analysis was uncoupled, and the heat source was modeled as the Goldak double ellipsoid [81]. A unique methodology in this work was the implementation of stress relaxation, where the stress and strains were reset to 0 when the temperature exceeded a defined stress relaxation temperature. Different values of relaxation temperature were investigated, and it was reported that the absence of relaxation effects resulted in over-prediction of distortion by more than 500%, and a simulated relaxation temperature of 690 °C matched measured values most closely (within 25%) [85].

Heigel et al. [71] modeled directed energy deposition (DED) of Ti–6Al–4V using the Goldak double ellipsoid heat source model [81]. The stress relaxation technique described by Denglinger et al. [85] was employed. Free and forced convection as boundary conditions were investigated in the simulations, and RS predictions were compared to experimental results. The forced convection model matched most closely to experimental results [71].

Wang et al. [86] used neutron diffraction to measure RSs in Inconel 625 manufactured by DED, and they compared measurements to a finite element model of the same process. The thermo-mechanical analysis was coupled, employing the Goldak double ellipsoid heat source model [81], with radiation and convection boundary conditions. The heat treatment applied to the sample may have relieved RSs, leading to an error in the computation of the residual strains and stresses [86].

EBAM of Inconel 718 was modeled by Prabhakar et al. [87]. In order to save computation time, the uncoupled thermo-mechanical analysis assumed the heat transfer of the process to be uniform across the entire layer, due to the rapid process, and radiation effects were also ignored. Distortions caused by RSs were qualitatively compared to experiment, and they were observed to be in agreement [87].

Denlinger et al. [88] developed a finite element model to predict RS formation in Inconel 718 produced by laser PBF processes. The heat source was modeled with the Goldak double ellipsoid model [81], but the thermal boundary conditions were treated in a way that accounted for heat source–particle interactions; the thermal conductivity of the powder, *k_p_*, was calculated as follows [88]:

(14) $$k_{p}=k_{f}\left[\left(1-\sqrt{1-\emptyset }\right)\left(1+\emptyset \frac{k_{r}}{k_{f}}\right)+\sqrt{1-\emptyset }\left(\frac{2}{1-\frac{k_{f}}{k_{s}}}\left(\frac{2}{1-\frac{k_{f}}{k_{s}}}\ln\frac{k_{s}}{k_{f}}-1\right)+\frac{k_{r}}{k_{f}}\right)\right]\;,$$

where *k_f_* is the thermal conductivity of the argon gas surrounding the particles, *ϕ* is the fractional porosity of the powder bed, *k_s_* is the conductivity of the solid, and *k_r_* is the heat transfer of the radiation between the individual particles [88], calculated as follows:

(15) $$k_{r}=\frac{4}{3}\sigma T^{3}D_{p}\;,$$

where *D_p_* is the average diameter of the particles, *σ* is the Stefan–Boltzmann constant, and *T* is the temperature. The emissivity of the powder is also calculated as follows [88]:

(16) $$\varepsilon_{p}=A_{H}\varepsilon_{H}+\left(1-A_{H}\right)\varepsilon_{s}\;,$$

where *A_H_* is the porous area fraction of the powder surface, *ε_H_* is the emissivity of the powder surface vacancies, and *ε_s_* is the emissivity of the solid, defined as follows [88]:

(17) $$A_{H}=\frac{0.908\;\emptyset^{2}}{1.908\;\emptyset^{2}-2\emptyset +1}\;,$$

(18) $$\varepsilon_{H}=\frac{\varepsilon_{s}\left[2+3.082{\left(\frac{1-\emptyset }{\emptyset }\right)}^{2}\right]}{\varepsilon_{s}\left[1+3.082{\left(\frac{1-\emptyset }{\emptyset }\right)}^{2}\right]+1}.$$

A mesh coarsening strategy was also implemented for computation time considerations, which allowed elements below the deposited layer to merge as the heat sources moved in the build direction. The thermal and mechanical analyses were uncoupled, and the distortion predictions caused by RSs agreed strongly with measurements (maximum error of 5%) [88].

Zhao et al. [89] modeled RS formation in direct metal laser sintering of Ti–6Al–4V with a coupled thermo-mechanical analysis. The heat source was modeled as two distributions for comparison. Firstly, a uniform heating pattern was used, given as follows [89]:

(19) $$Q=\;\frac{P}{\pi r^{2}}.$$

Next, a semi-spherical power distribution model was used, given as follows [89]:

(20) $$Q=\;\frac{3P}{2\pi r^{2}}\sqrt{\left|1-\frac{{\left(x-x_{0}\right)}^{2}}{r^{2}}-\frac{{\left(y-y_{0}\right)}^{2}}{r^{2}}\right|}\;,\;\;\;\;\;\;\;\;\;\mathrm{for}\;\left|x-x_{0}\right|<r\;\mathrm{and}\;\;\left|y-y_{0}\right|<r,\;$$

where *x*_0_ and *y*_0_ are coordinates of the laser spot center. The properties of the powder were estimated from the density, *ρ*_0_, heat capacity, *c_p_*_0_, and thermal conductivity, *k*_0_, of the solid material as follows [89]:

(21) $$\frac{\rho }{\rho_{0}}=1-\emptyset \;,$$

(22) $$\frac{c_{p}}{c_{p0}}=1-\emptyset \;,$$

(23) $$\frac{k}{k_{0}}=\frac{1-\emptyset }{1+11\;\emptyset^{2}}.$$

The emissivity of the powder was also assumed, as discussed above [88]. Calculated residual stresses in Reference [89] were not compared to experimental results. 

## 5. Residual Stress-Induced Distortion Prevention and Compensation

### 5.1. Approaches to Prevent Deflection

As discussed previously, adjusting AM process parameters can reduce RSs, but it may not completely eliminate them from forming; therefore, strategies exist to take advantage of the distortions induced by RSs to achieve accurate part dimensions. Mukherjee et al. [90] showed, analytically, that the process variables from AM influence thermal strains (and, thus, thermal stresses), to guide researchers investigating the mitigation of part distortion. It was shown that low heat input can reduce thermal strains, the combined effect of a decrease in laser power and layer height can reduce the distortion, and the combination of a decrease in laser power and an increase in scanning speed can reduce the thermal strains.

**Figure 22:**
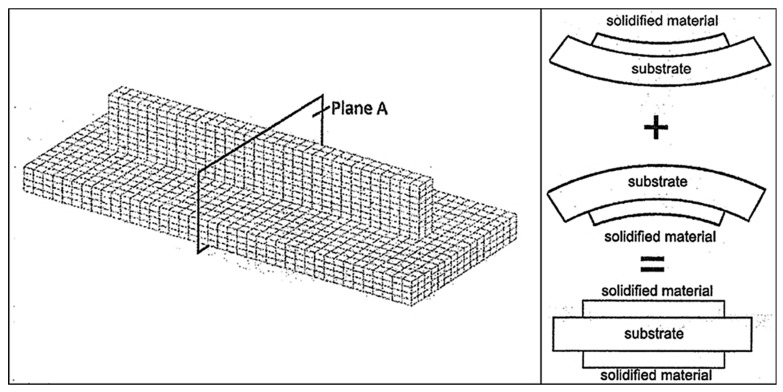
Desired geometry with cross-sectional plane for residual stress measurements (**left**), and proposed concept (**right**) by Denlinger and Michaleris—upward and downward distortion mitigated by a balance of sacrificial material [91].

**Figure 23:**
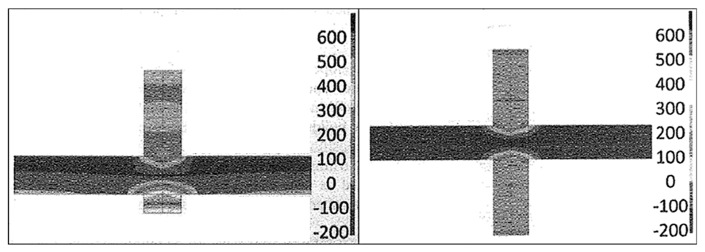
Residual stress distribution with only four sacrificial layers added (**left**) and with 12 balanced layers (**right**); the balanced sacrificial material yields a uniform distribution, reducing the distortion of the part [91].

Denlinger and Michaleris [91] investigated three distortion mitigation techniques in EBAM with wire feedstock of Ti–6Al–4V. Three distortion mitigation techniques were investigated. Firstly, the part was heated after the deposition to relax the thermal stresses; the other two methods involved the deposition of additional material across the neutral axis of the build, extending beyond the part geometry, to be machined after completion, either after the completion of each layer or after the completion of all layers. These methods were examined with finite element analysis, and the most successful was implemented into experiment. It was determined that depositing additional material after the completion of each layer was the most successful, with 91% of the bending distortion eliminated (see Figure 22 and Figure 23) [91].

**Figure 24:**
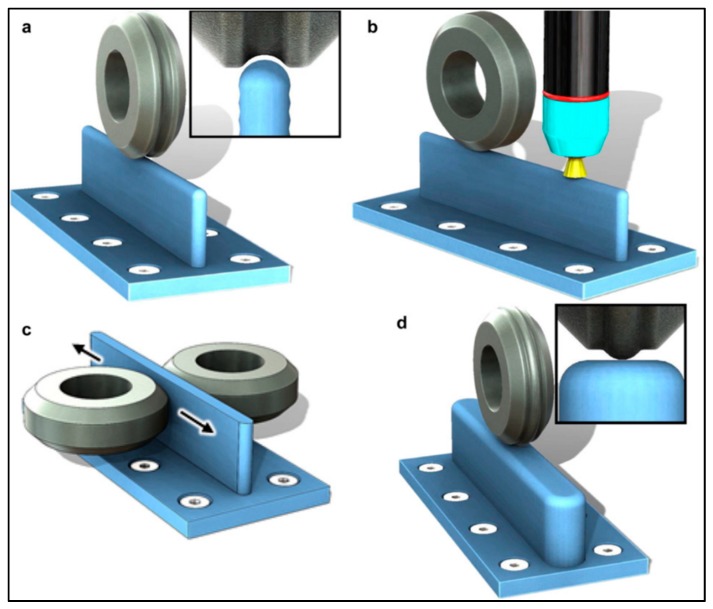
Investigations of bulk deformation processes (rolling) applied to AM components during the build process as a slotted roller in the build direction (**a**), flat rolling ahead of the heat source in the build direction (**b**) or in the transverse direction (**c**), and an inverted profile roller for thick regions (**d**) [92].

Colegrove et al. [92] investigated bulk deformation processes applied during the AM process for property, RS, and distortion control. Rolling was applied to the WAAM of Ti–6Al–4V to apply plastic deformation to relax the RSs formed. Depending on the orientation of the roller with respect to the part (shown in Figure 24), the distortion and RS could be nearly eliminated from about 550 MPa to less than 200 MPa [92].

Aggarangsi and Beuth [93] investigated the effects of localized pre-heating in AM for reducing the RSs generated. The researchers mentioned that uniform pre-heating was used to reduce RSs in small parts, but it was impractical for very large parts; thus, they proposed a localized pre-heating method. The two proposed methods involved a second heat source (lower power) in front of or behind the melt pool, or the pre-heating of the top surface to a predetermined temperature. Uncoupled thermal–structural simulations were used to investigate the effects on RS and part distortion in AISI 304 stainless steel. It was determined that the additional heat source following or leading the primary beam did not significantly reduce the thermal gradient and RSs. However, pre-heating the top-most layer to 673 K did reduce the maximum stress by 18% [93].

In a study by Stucker et al. [94], RapidSteel 2.0 and LaserForm ST-100 (specialty steels) parts were fabricated via SLM, and the process parameters during the post-AM furnace/infiltration stage were investigated for their effect on final part geometry. It was determined that the LaserForm ST-100 was sensitive to the temperature ramp rate in the furnace, and it had more uniform shrinkage between the axes and better feature definition (sharper corners); however, it had much larger absolute shrinkage than the RapidSteel 2.0, which was more sensitive to the amount of infiltrant used. It was suggested that a furnace cycle, which heated the part very rapidly, held it for the least amount of time necessary for infiltration, and then cooled the part very slowly, would minimize shrinkage [94].

Krol et al. [76] compared simulation results with neutron diffraction-measured RSs in AlSi12. Process parameters were investigated for their influence on RS formation, including pre-heating the specimen, increasing the scanning velocity, and changing the support pattern from a block to an optimized pattern (with respect to the RSs simulated). The stress measurements indicated that the optimized support pattern consistently resulted in a lower RS state (about 70 GPa vs. about 10 GPa in some instances). Interestingly, the pre-heated (200 °C) specimens had higher tensile stresses than the room-temperature specimens, and the increase in scanning velocity reduced the RSs formed [76].

The effect of inter-layer dwell time on RS formation in Inconel 718 and Ti–6Al–4V by a laser-based directed energy deposition was investigated by Denlinger et al. [95]. It was determined that the dwell time between layers had a significant effect on the formation of RSs. The dwell time serves to allow additional cooling during the AM process. It was found that, for Inconel 718, an increase in dwell time from 0 to 40 s showed very small changes in distortion and magnitude of RSs; however, for the titanium alloy, the longer dwell times increased distortion by as much as 54%, and RSs by as much as 122%. Distortion measurements were made using a coordinate measurement machine (CMM), and RS was measured with the hole-drilling method [95]. 

Vastola et al. [73] explored a finite element simulation of Ti–6Al–4V alloy manufactured by electron beam melting to investigate the effects of beam size, beam power, scanning speed, and powder bed temperature on RS development. A small beam size was found to generate larger RSs within a smaller heat-affected zone (HAZ), and larger beam sizes had a larger HAZ with a more uniform stress distribution. The beam power was increased by 20%, and the HAZ was observed to increase by 15% and, thus, increase the distribution of generated stresses. The scanning speed was seen to affect the depth of the HAZ; lower scan speeds had a deeper HAZ and deeper thermal gradients, thereby leading to deeper RS generation. Finally, an increase in powder bed temperature was seen to reduce the magnitudes of the RSs more than the other parameters [73].

**Figure 25:**
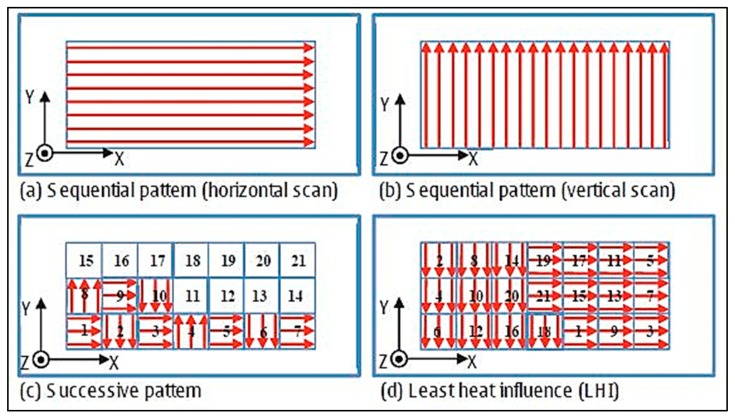
Different scan patterns investigated for residual stress formation in SLM [96].

In studies by Li et al. [96] and Li et al. [97], a multiscale finite element model was developed to accurately predict part distortion caused by RS formation in SLM processes. The simulation was compared against experimental data for an iron-based powder. The simulation started with a micro-scale model of the heat source–powder particle interaction, to obtain the temperature distribution of the molten material. An equivalent heat flux was developed from the temperature data in the micromodel and applied to a meso-scale model to obtain a RS field. Finally, the stress field was fed into a macro-scale model to obtain part distortion with different scan strategies. The simulation results were compared with experimental data and found to agree. Furthermore, the different scan strategies revealed that a successive island strategy resulted in lower RS formation than the least heat influence island strategy (see Figure 25) [96,97].

### 5.2. Corrective Design to Mitigate Residual Stresses in AM

**Figure 26:**
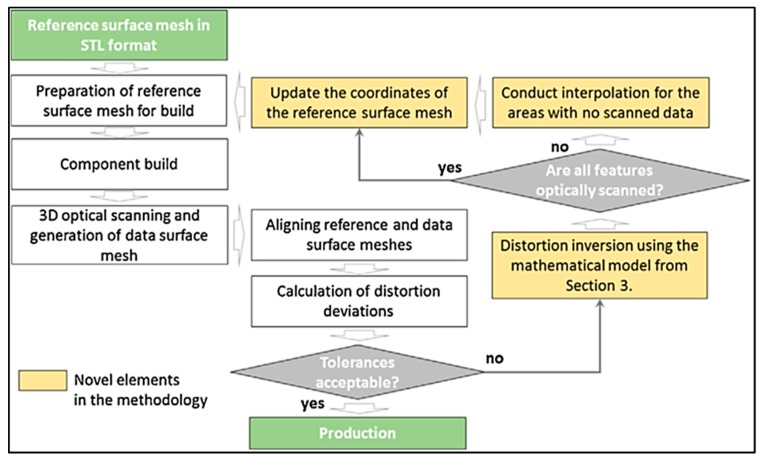
Workflow of proposed distortion prevention method in AM [98].

Afazov et al. [98] developed a method to compensate for the RSs and the subsequent distortions in AM parts. The proposed method used a mathematical model to pre-distort the CAD geometry based on a 3D scan of the distorted, as-built part. The workflow, shown in Figure 26, firstly prints the part from the CAD file, scans the part, and compares the 3D scan data with the CAD geometry; then, it inverts the distortion (while interpolating areas where measurement data are not available) and re-creates a corrected surface mesh for printing. Using this distortion-compensation technique on an impeller geometry (110 mm diameter, 40 mm height, with turbine blade height of 80 mm), Inconel 718 parts fabricated in laser powder bed fusion were successful with tolerances of ±65 μm [98].

Afazov et al. [99] also implemented a distortion correction, to take advantage of the generated RSs, in order to obtain the correct part geometry. SLM was modeled with finite element analysis using Ti–6Al–4V powder. The distortion was predicted, and the displacements of the mesh were inverted. The coordinates of the mesh were updated based on the inverted distortion prediction. It was shown that the non-corrected geometry had distortion of ±200 m, and the corrected geometry from the FEA resulted in distortions of ±45 m [99].

**Figure 27:**
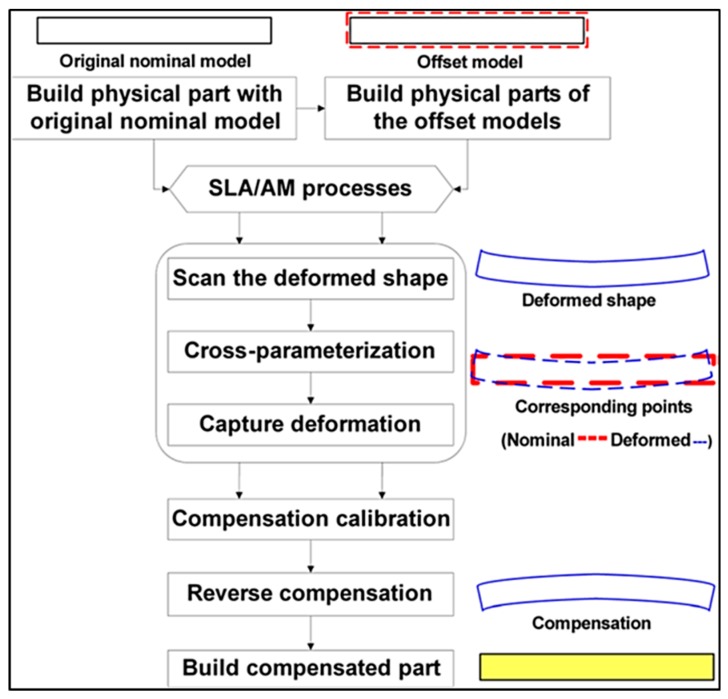
Workflow of the proposed distortion prevention technique [100].

Similar to the work of Afazov et al. [98] and Afazov et al. [99], Xu et al. [100] developed a computational framework to compensate for the distortion in AM processes, to manufacture dimensionally/geometrically accurate parts. The developed process is shown in Figure 27. The part is additively manufactured from a CAD model, and the as-built (distorted) geometry is measured with a CMM or 3D scanner. The distorted geometry is compared to the original CAD model, and a new, “corrected” CAD model is generated by inverting the displacements between the geometry and the CAD model. The new CAD model, then, has dimensions that, when printed, would distort into the correct final geometry. The authors reported an improvement in shape deformation of 55% when the compensated framework was implemented [100].

**Figure 28:**
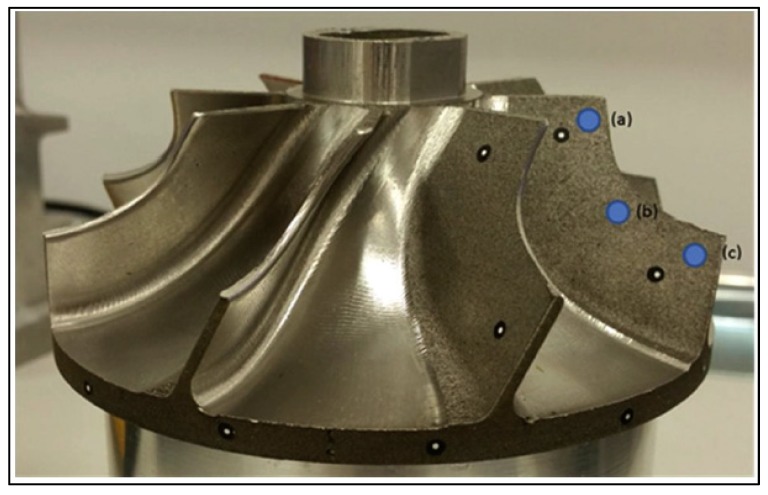
Printed impeller discussed in Yaghi et al. [101]. The measured surface RSs for (**a**,**b**) were around 550 and 250 MPa, respectively; (**c**) had an RS of 150 MPa about 0.5 mm below the surface.

In work by Yaghi et al. [101], a method for mitigating distortion in AM parts was presented. Two stainless-steel 316 impellers of 48.6 mm in height, with the largest diameter being 109 mm, were manufactured via a laser powder bed fusion AM process and then machined to the final geometry, as shown in Figure 28. Measurements of distortion and RSs were taken on the parts using optical measurements and the hole-drilling method combined with the contour method. In Figure 28, the surface RSs changed from ~550 to ~250 MPa from (a) to (b). Point (c) had residual stresses of ~150 MPa about 0.5 mm below the surface. A finite element model was developed to predict distortion, which was validated with the measured data. The predicted distortions were applied to the finite element model in the negative direction, and the surface mesh was re-mapped, resulting in a pre-distorted CAD model for printing. Using the distortion compensation method, the part distortion was reduced from 200 μm to less than 100 μm [101]. 

It is important to note that the distortion prevention techniques in which the CAD geometry is altered, such that the distortion during manufacture results in an accurate geometry, as seen in References [98,99,100,101], only enables dimensional accuracy; the RSs still remain in the parts. While the final geometry of the part is correct, the fatigue life and structural integrity of the part might still be affected [10]. According to Li et al. [28], the most common approach currently employed to reduce the residual stresses in AM processes is to pre-heat the feed stock or substrate to decrease the thermal gradients during manufacture. Furthermore, a post-manufacturing heat treatment of AM parts was shown to reduce the dislocation density and, thus, type III RSs.

## 6. Conclusions and Future Works

AM is a growing technology, but there is a huge demand for RS and part distortion prevention in additively manufactured components, as one of the most appealing qualities of AM is the ability to produce complex near-net shape geometries usually faster than most other techniques. Any distortion in precision parts can be catastrophic in use, and RSs can negatively impact the fatigue life and other mechanical performance characteristics. It was demonstrated that the microstructure of various material systems can change during AM processes, and inhomogeneous microstructure evolution, as well as non-uniform phase transformations, can generate RSs within the material. In this regard, stress-relieving heat treatments can help create a more uniform and stress-free microstructure.

As discussed, there are a number of methods for measuring RS, each with advantages and disadvantages, different spatial resolutions, and capabilities regarding the size of the part. There are also a variety of AM techniques and variable parameters available, each of which was shown to induce RSs in the part, exceeding the yield strength of a material in some cases. It was shown that track length, island size, laser power, and scanning speed need to be optimized to reduce the generated RS formation. Much work was conducted to explore the effects of process parameters on RS formation, both experimentally and computationally.

In this paper, different AM technologies were described, and recent investigations on RS formation were reported. As governed by the temperature gradient mechanism, compressive stresses typically form in the center of the part, and tensile stresses form on the top layers. The largest magnitudes of RSs are observed in the direction of the scan line. Small, successive scan islands result in lower RSs than directional scanning. Faster scanning was observed to result in lower RSs than slower scanning, due to the energy input that goes with the scanning speed. The dwell time between layers affects the RSs formed, with large dwell times causing larger RSs. Finally, pre-heating the baseplate prior to build can decrease the thermal gradients and lower the magnitudes of RSs. The EBAM process was shown generate RSs lower in magnitude, compared to SLM, but SLM remains one of the most common technologies due to its affordable and simple (no vacuum chamber) set-up.

The benefits of computational models of AM processes include the rapid and inexpensive investigations of process parameters on RS, but the downside is the computational burden of the simulations, as well as the correct capture of the physics and the validity of the assumptions made. Experimental investigations are, of course, desirable, but they are expensive and time-consuming. The research community needs a physically accurate and computationally inexpensive AM process simulation. Another important and often neglected source of RS formation is the microstructure evolution; this impacts the fundamental material properties, and it plays a direct role in performance, but it is often missed in the simulations. 

In addition to a computational framework to accurately capture the physics of AM processes, further experimental investigations into residual stress prevention techniques must be conducted, not just the distortion prevention techniques. While the geometry is preserved in the distortion prevention methods, the RSs (which are the source of the distortion) remain in the part, affecting the fatigue life and structural integrity of the components. Furthermore, in order to more accurately capture the development and evolution of RSs in AM by computer simulations, micro-RS contributions need to be superimposed to the current macro-scale frameworks. In this regard, strong coupling of phase field schemes to the current formalisms mentioned in detail is a must in any robust AM modeling framework, in order to account for dendritic growth and other solidification phenomena and the associated expansions and contractions of the semi-solid and re-solidified regions around the melt pool. Additionally, considering the effects of anisotropy of elastic properties in the semi-solid and re-solidified regions in the simulation of RS development is yet to be studied.

## References

[1] Attaran M. (2017). The rise of 3-D printing: The advantages of additive manufacturing over traditional manufacturing. Bus. Horiz..

[2] Allen J. (2006). An Investigation into the Comparative Costs of Additive Manufacture vs. Machine from Solid for Aero Engine Parts. Cost Effective Manufacture via Net-Shape Processing.

[3] Thomas D. (2016). Costs, Benefits, and Adoption of Additive Manufacturing: A Supply Chain Perspective. Int. J. Adv. Manuf. Technol..

[4] Thomas D.S., Gilbert S.W. (2014). Costs and Cost Effectiveness of Additive Manufacturing. NIST Spec. Publ..

[5] Withers P.J., Bhadeshia H.K.D.H. (2001). Residual stress. Part 2—Nature and origins. Mater. Sci. Technol..

[6] Saphronov V.A., Khmyrov R.S., Grigoriev S.N., Gusarov A.V. (2016). Distortions and Residual Stresses at Layer-by-Layer Additive Manufacturing by Fusion. J. Manuf. Sci. Eng..

[7] Roy T.D., Wei H.L., Zuback J.S., Mukherjee T., Elmer J.W., Milewski J.O., Beese A.M., Wilson-Heid A., De A., Zhang W. (2018). Additive manufacturing of metallic components—Process, structure and properties. Prog. Mater. Sci..

[8] Buchbinder D., Meiners W., Pirch N., Wissenbach K., Schrage J. (2014). Investigation on reducing distortion by preheating during manufacture of aluminum components using selective laser melting. J. Laser Appl..

[9] Li C., Liu J.F., Fang X.Y., Guo Y.B. (2017). Efficient predictive model of part distortion and residual stress in selective laser melting. Addit. Manuf..

[10] Withers P.J. (2007). Residual stress and its role in failure. Rep. Prog. Phys..

[11] Zhang J., Wang X., Paddea S., Zhang X. (2016). Fatigue crack propagation behaviour in wire + arc additive manufactured Ti-6Al-4V: Effects of microstructure and residual stress. Mater. Des..

[12] Withers P.J., Bhadeshia H.K.D.H. (2001). Residual stress. Part 1—Measurement techniques. Mater. Sci. Technol..

[13] Rossini N.S., Dassisti M., Benyounis K.Y., Olabi A.G. (2012). Methods for Measuring Residual Stresses in Components. Mater. Des..

[14] Hrabe N., Gnäupel-herold T., Quinn T. (2017). Fatigue properties of a titanium alloy (Ti–6Al–4V) fabricated via electron beam melting (EBM): Effects of internal defects and residual stress. Int. J. Fatigue.

[15] Dieter G. (1961). Metallurgy and Metallurgical Engineering Series.

[16] Todd R.I., Boccaccini A.R., Sinclair R., Yallee R.B., Young R.J. (1999). Thermal residual stresses and their toughening effect in Al_2_O_3_ platelet reinforced glass. Acta Mater..

[17] Shorr B.F. (2015). Thermal Integrity in Mechanics and Engineering.

[18] Philpot T.A. (2012). Mechanics of Materials: An Integrated Learning System.

[19] Herzog D., Seyda V., Wycisk E., Emmelmann C. (2016). Additive manufacturing of metals. Acta Mater..

[20] LeBrun T., Nakamoto T., Horikawa K., Kobayashi H. (2015). Effect of retained austenite on subsequent thermal processing and resultant mechanical properties of selective laser melted 17-4 PH stainless steel. Mater. Des..

[21] Murr L.E., Martinez E., Hernandez J., Collins S., Amato K.N., Gaytan S.M., Shindo P.W. (2012). Microstructures and properties of 17-4 PH stainless steel fabricated by selective laser melting. J. Mater. Res. Technol..

[22] Uhlmann E., Kersting R., Klein T.B., Cruz M.F., Borille A.V. (2015). Additive Manufacturing of Titanium Alloy for Aircraft Components. Procedia CIRP.

[23] Nickels L. (2016). Additive Manufacturing: A User’s Guide.

[24] Frazier W.E. (2014). Metal additive manufacturing: A review. J. Mater. Eng. Perform..

[25] Bikas H., Stavropoulos P., Chryssolouris G. (2016). Additive manufacturing methods and modelling approaches: A critical review. Int. J. Adv. Manuf. Technol..

[26] Kruth J.P. (1991). Material Incress Manufacturing by Rapid Prototyping Techniques. CIRP Ann..

[27] Gong X., Anderson T., Chou K. Review on powder-based electron beam additive manufacturing technology. Proceedings of the ASME/ISCIE 2012 International Symposium on Flexible Automation.

[28] Li C., Liu Z.Y., Fang X.Y., Guo Y.B. (2018). Residual Stress in Metal Additive Manufacturing. Procedia CIRP.

[29] Mercelis P., Kruth J.P. (2006). Residual stresses in selective laser sintering and selective laser melting. Rapid Prototyp. J..

[30] Kruth J.P., Froyen L., van Vaerenbergh J., Mercelis P., Rombouts M., Lauwers B. (2004). Selective laser melting of iron-based powder. J. Mater. Process. Technol..

[31] Gusarov A.V., Pavlov M., Smurov I. (2011). Residual stresses at laser surface remelting and additive manufacturing. Phys. Procedia.

[32] Wu A.S., Brown D.W., Kumar M., Gallegos G.F., King W.E. (2014). An Experimental Investigation into Additive Manufacturing- Induced Residual Stresses in 316L Stainless Steel. Metall. Mater. Trans. A.

[33] Lu Y., Wu S., Gan Y., Huang T., Yang C., Junjie L., Lin J. (2015). Study on the microstructure, mechanical property and residual stress of SLM Inconel-718 alloy manufactured by differing island scanning strategy. Opt. Laser Technol..

[34] Liu Y., Yang Y., Wang D. (2016). A study on the residual stress during selective laser melting (SLM) of metallic powder. Int. J. Adv. Manuf. Technol..

[35] Van Belle L., Vansteenkiste G., Boyer J.C. (2013). Investigation of Residual Stresses Induced during the Selective Laser Melting Process. Key Eng. Mater..

[36] Xiaoquing W., Kevin C. (2013). Residual Stress in Metal Parts Produced by Powder-Bed Additive Manufacturing Processes. J. Chem. Inf. Model..

[37] Cottam R., Wang J., Luzin V. (2014). Characterization of microstructure and residual stress in a 3D H13 tool steel component produced by additive manufacturing. J. Mater. Res..

[38] Cao J., Gharghouri M.A., Nash P. (2016). Finite-element analysis and experimental validation of thermal residual stress and distortion in electron beam additive manufactured Ti-6Al-4V build plates. J. Mater. Process. Technol..

[39] Olabi A.G., Hashimi M.S.J. (1996). Stress relief procedures for low carbon steel (1020) welded components. J. Mater. Process. Technol..

[40] Tebedge N., Alpsten G., Tall L. (1973). Residual-stress Measurement by the Sectioning Method. Exp. Mech..

[41] Cullity B.D., Stock S.R. (2001). Elements of X-ray Diffraction.

[42] Mishurova T., Cabeza S., Artzt K., Haubrich J., Klaus M., Genzel C., Requena G., Bruno G. (2017). An assessment of subsurface residual stress analysis in SLM Ti–6Al–4V. Materials.

[43] Yan J.J., Zheng D.L., Li H.X., Jia X., Sun J.F., Li Y.L., Yan M. (2017). Selective laser melting of H13: Microstructure and residual stress. J. Mater. Sci..

[44] Santisteban J.R., Edwards L., Steuwer A., Withers P.J. (2001). Time-of-flight neutron transmission diffraction. J. Appl. Crystallogr..

[45] Tsui T.Y., Oliver W.C., Pharr G.M. (1996). Influences of stress on the measurement of mechanical properties using nanoindentation: Part 1. Experimental studies in an aluminum alloy. J. Mater. Res..

[46] Bolshakov A., Oliver W.C., Pharr G.M. (1996). Inlfuences of stress on the measurement of mechanical properties using nanoindentation: Part II. Finite Element Simulations. J. Mater. Res..

[47] Suresh S., Giannakopoulos A.E. (1998). A new method for estimating residual stresses by instrumented sharp indentation. Acta Mater..

[48] Ghidelli M., Sebastiani M., Collet C., Guillemet R. (2016). Determination of the elastic moduli and residual stresses of freestanding Au-TiW bilayer thin films by nanoindentation. Mater. Des..

[49] EHerbert G., Oliver W.C., de Boer M.P., Pharr G.M. (2009). Measuring the elastic modulus and residual stress of freestanding thin films using nanoindentation techniques. J. Mater. Res..

[50] Wang L., Bei H., Gao Y.F., Lu Z.P., Nieh T.G. (2011). Effect of residual stresses on the hardness of bulk metallic glasses. Acta Mater..

[51] Wang X., Gong X., Chou K. (2015). Scanning Speed Effect on Mechanical Properties of Ti-6Al-4V Alloy Processed by Electron Beam Additive Manufacturing. Procedia Manuf..

[52] Li W., Liu W., Qi F., Chen Y., Xing Z. (2019). Determination of micro-mechanical properties of additive manufactured alumina ceramics by nanoindentation and scratching. Ceram. Int..

[53] Gong X., Lydon J., Cooper K., Chou K. Microstructural Analysis and Nanoindentation Characterization of Ti–6Al–4V Parts from Electron Beam Additive Manufacturing. Proceedings of the ASME 2014 International Mechanical Engineering Congress and Exposition.

[54] Hoye N., Li H.J., Cuiuri D., Paradowska A.M. (2014). Measurement of Residual Stresses in Titanium Aerospace Components Formed via Additive Manufacturing. Mater. Sci. Forum.

[55] Ding J., Colegrove P., Mehnen J., Ganguly S., Almeida P.S., Wang F., Williams S. (2011). Thermo-mechanical analysis of Wire and Arc Additive Layer Manufacturing process on large multi-layer parts. Comput. Mater. Sci..

[56] Colegrove P.A., Coules H.E., Fairman J., Martina F., Kashoob T., Mamash H., Cozzolino L.D. (2013). Microstructure and residual stress improvement in wire and arc additively manufactured parts through high-pressure rolling. J. Mater. Process. Technol..

[57] An K., Yuan L., Dial L., Spinelli I., Stoica A.D., Gao Y. (2017). Neutron residual stress measurement and numerical modeling in a curved thin-walled structure by laser powder bed fusion additive manufacturing. Mater. Des..

[58] Brice C.A., Hofmeister W.H. (2013). Determination of bulk residual stresses in electron beam additive-manufactured aluminum. Metall. Mater. Trans. A Phys. Metall. Mater. Sci..

[59] Simson T., Emmel A., Dwars A., Böhm J. (2017). Residual stress measurements on AISI 316L samples manufactured by selective laser melting. Addit. Manuf..

[60] Ahmad B., van der Veen S.O., Fitzpatrick M.E., Guo H. (2018). Residual stress evaluation in selective-laser-melting additively manufactured titanium (Ti–6Al–4V) and inconel 718 using the contour method and numerical simulation. Addit. Manuf..

[61] Knowles C.R., Becker T.H., Tait R.B. (2012). Residual Stress measurements and structural integrity implications for selective laser melted Ti-6Al-4V. S. Afr. J. Ind. Eng..

[62] Schoinochoritis B., Chantzis D., Salonitis K. (2017). Simulation of metallic powder bed additive manufacturing processes with the finite element method: A critical review. Proc. Inst. Mech. Eng. Part B J. Eng. Manuf..

[63] Zohdi T.I. (2015). Modeling and simulation of cooling-induced residual stresses in heated particulate mixture depositions in additive manufacturing. Comput. Mech..

[64] Megahed M., Mindt H., Dri N.N., Duan H., Desmaison O. (2016). Metal additive-manufacturing process and residual stress modeling. Integr. Mater. Manuf. Innov..

[65] Luo Z., Zhao Y. (2018). A survey of fi nite element analysis of temperature and thermal stress fi elds in powder bed fusion Additive Manufacturing. Addit. Manuf..

[66] Ganeriwala R.K. (2015). Multiphysics Modeling of Selective Laser Sintering/Melting.

[67] Ganeriwala R., Zohdi T.I. (2014). Multiphysics modeling and simulation of selective laser sintering manufacturing processes. Procedia CIRP.

[68] Roberts I.A., Wang C.J., Esterlein R., Stanford M., Mynors D.J. (2009). A three-dimensional finite element analysis of the temperature field during laser melting of metal powders in additive layer manufacturing. Int. J. Mach. Tools Manuf..

[69] Incropera F.P., Lavine A.S., Bergman T.L., DeWitt D.P. (2007). Fundamentals of Heat and Mass Transfer.

[70] Labudovic M., Hu D., Kovacevic R. (2003). A Three Dimensional Model For Direct Laser Metal Powder Deposition and Rapid Prototyping. J. Mater. Sci..

[71] Heigel J.C., Michaleris P., Reutzel E.W. (2015). Thermo-mechanical model development and validation of directed energy deposition additive manufacturing of Ti–6Al–4V. Addit. Manuf..

[72] Ghosh S., Choi J. (2006). Modeling and Experimental Verification of Transient/Residual Stresses and Microstructure Formation in Multi-Layer Laser Aided DMD Process. J. Heat Transf..

[73] Vastola G., Zhang G., Pei Q.X., Zhang Y. (2016). Controlling of residual stress in additive manufacturing of Ti6Al4V by finite element modeling. Addit. Manuf..

[74] Zaeh M.F., Branner G., Krol T.A. (2009). A three dimensional FE-model for the investigation of transient physical effects in Selective Laser Melting. Innovative Developments in Design and Manufacturing.

[75] Zaeh M.F., Branner G. (2010). Investigations on residual stresses and deformations in selective laser melting. Prod. Eng..

[76] Krol T.A., Seidel C., Schilp J., Hofmann M., Gan W., Zaeh M.F. (2013). Verification of structural simulation results of metal-based additive manufacturing by means of neutron diffraction. Phys. Procedia.

[77] Gu D., He B. (2016). Finite element simulation and experimental investigation of residual stresses in selective laser melted Ti–Ni shape memory alloy. Comput. Mater. Sci..

[78] Lampa C., Kaplan A.F.H., Powell J., Magnusson C. (1997). An analytical thermodynamic model of laser welding. J. Phys. D Appl. Phys..

[79] Myhr O.R., Klokkehaug S., Grong O., Fjaer H.G., Kluken A.O. (1998). Modeling of microstructure evolution, residual stresses and distortions in 6082-T6 aluminum weldments. Weld. J. N. Y..

[80] Denlinger E.R., Irwin J., Michaleris P. (2014). Thermomechanical Modeling of Additive Manufacturing Large Parts. J. Manuf. Sci. Eng..

[81] Goldak J., Chakravarti A., Bibby M. (1984). A new finite element model for welding heat sources. Metall. Trans. B.

[82] Chae H.M. (2013). A Numerical and Experimental Study for Resdiual Stress Evolution in Low Alloy Steel during Laser Aided Additive Manufacturing Process.

[83] Parry L., Ashcroft I.A., Wildman R.D. (2016). Understanding the effect of laser scan strategy on residual stress in selective laser melting through thermo-mechanical simulation. Addit. Manuf..

[84] Li C., Liu J.F., Guo Y.B. (2016). Prediction of Residual Stress and Part Distortion in Selective Laser Melting. Procedia CIRP.

[85] Denlinger E.R., Heigel J.C., Michaleris P. (2015). Residual stress and distortion modeling of electron beam direct manufacturing Ti-6Al-4V. Proc. Inst. Mech. Eng. Part B J. Eng. Manuf..

[86] Wang Z., Denlinger E., Michaleris P., Stoica A.D., Ma D., Beese A.M. (2017). Residual stress mapping in Inconel 625 fabricated through additive manufacturing: Method for neutron diffraction measurements to validate thermomechanical model predictions. Mater. Des..

[87] Prabhakar P., Sames W.J., Dehoff R., Babu S.S. (2015). Computational modeling of residual stress formation during the electron beam melting process for Inconel 718. Addit. Manuf..

[88] Denlinger E.R., Gouge M., Irwin J., Michaleris P. (2017). Thermomechanical model development and in situ experimental validation of the Laser Powder-Bed Fusion process. Addit. Manuf..

[89] Zhao X., Iyer A., Promoppatum P., Yao S. (2017). Numerical modeling of the thermal behavior and residual stress in the direct metal laser sintering process of titanium alloy products. Addit. Manuf..

[90] Mukherjee T., Manvatkar V., Debroy T. (2017). Mitigation of thermal distortion during additive manufacturing. Scr. Mater..

[91] Denlinger E.R., Michaleris P. (2017). Mitigation of distortion in large additive manufacturing parts. Proc. Inst. Mech. Eng. Part B J. Eng. Manuf..

[92] Colegrove P.A., Donoghue J., Martina F., Gu J., Prangnell P., Hönnige J. (2017). Application of bulk deformation methods for microstructural and material property improvement and residual stress and distortion control in additively manufactured components. Scr. Mater..

[93] Aggarangsi P., Beuth J.L. Localized preheating approaches for reducing residual stress in additive manufacturing. Proceedings of the Solid Freeform Fabrication Symposium.

[94] Stucker B., Malhotra M., Qu X., Hardro P., Mohanty N. RapidSteel Part Accuracy. Proceedings of the International Solid Freeform Fabrication Symposium.

[95] Denlinger E.R., Heigel J.C., Michaleris P., Palmer T.A. (2015). Effect of inter-layer dwell time on distortion and residual stress in additive manufacturing of titanium and nickel alloys. J. Mater. Process. Technol..

[96] Li C., Fu C.H., Guo Y.B., Fang F.Z. (2015). Fast Prediction and Validation of Part Distortion in Selective Laser Melting. Procedia Manuf..

[97] Li C., Fu C.H.H., Guo Y.B.B., Fang F.Z.Z. (2016). A multiscale modeling approach for fast prediction of part distortion in selective laser melting. J. Mater. Process. Technol..

[98] Afazov S., Okioga A., Holloway A., Denmark W., Triantaphyllou A., Smith S.A., Bradley-Smith L. (2017). A methodology for precision additive manufacturing through compensation. Precis. Eng..

[99] Afazov S., Denmark W.A.D., Toralles B.L., Holloway A., Yaghi A. (2017). Distortion prediction and compensation in selective laser melting. Addit. Manuf..

[100] Xu K., Kwok T.H., Zhao Z., Chen Y. (2017). A reverse compensation framework for shape deformation control in additive manufacturing. J. Comput. Inf. Sci. Eng..

[101] Yaghi A., Ayvar-Soberanis S., Moturu S., Bilkhu R., Afazov S. (2019). Design against distortion for additive manufacturing. Addit. Manuf..

